# Host–Guest Complexes of Cyclopentadienyl Iron Dicarbonyl (CpFe(CO)_2_) CO-Releasing Molecules with Cucurbit[7]uril

**DOI:** 10.1021/acs.organomet.4c00469

**Published:** 2025-03-26

**Authors:** Rodrigo P. Monteiro, Isabel B. Calhau, Ana C. Gomes, Ricardo F. Mendes, Filipe A. Almeida Paz, André D. Lopes, José P. Da Silva, Carlos C. Romão, Isabel S. Gonçalves, Martyn Pillinger

**Affiliations:** † CICECO - Aveiro Institute of Materials, Department of Chemistry, 426216 University of Aveiro, Campus Universitário de Santiago, Aveiro 3810-193, Portugal; ‡ Centre of Marine Sciences (CCMAR/CIMAR LA), and Department of Chemistry and Pharmacy, FCT, 70985 University of the Algarve, Faro 8005-039, Portugal; § Instituto de Tecnologia Química e Biológica António Xavier, Universidade Nova de Lisboa, Avenida da República (EAN), Oeiras 2780-157, Portugal

## Abstract

Iron­(II) cyclopentadienyl carbonyl complexes are promising as CO-releasing molecules (CORMs) for therapeutic applications. In common with other metallodrugs, the practical application of Fe-CORMs may require their conjugation with biocompatible carriers to improve their bioavailability and protect them from premature degradation. Here, we show that the CO-releasing properties of the complexes [CpFe­(CO)_2_Cl] (**1**) and [CpFe­(CO)_2_CH_2_CONH_2_] (**2**) are retained when noncovalently encapsulated within cucurbit[7]­uril (CB7), a well-established drug-enhancing excipient. The inclusion compounds were characterized in the solid-state by single-crystal and powder XRD, ATR-IR spectroscopy, Raman spectroscopy, TGA, and ^13^C­{^1^H} CP MAS NMR. In the crystal structure of **2**@CB7, there are two crystallographically independent [**2**@CB7] binary complexes that differ in the orientation of the guest molecules inside the CB cavity. High-resolution ESI-MS and ^1^H NMR studies verified the formation and stability of 1:1 **2**@CB7 inclusion complexes in an aqueous solution. In a physiological buffer, complex **2** is stable in the dark, but releases ca. 1.4 equiv of CO when irradiated with low-power cold white light, with a half-life (*t*_1/2_) of 19.2 ± 1.9 min. The photodecarbonylation behavior of the complexes is largely maintained in the inclusion compounds, with *t*_1/2_ of 10.0 ± 0.6 and 21.1 ± 1.9 min for encapsulated **1** and **2**.

## Graphical abstract

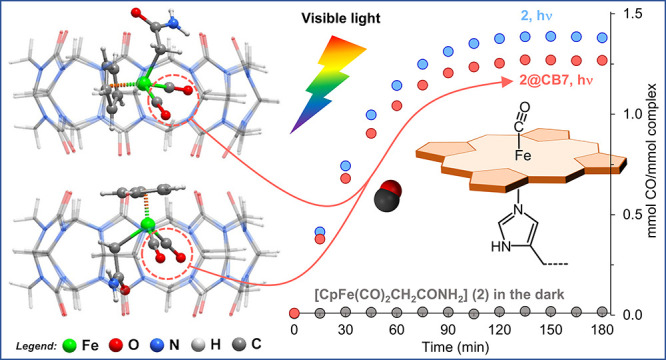

## Introduction

The field of medicinal inorganic chemistry has grown consistently over the past 50 years following the approval of cisplatin, *cis*-[PtCl_2_(NH_3_)_2_], as an anticancer drug in the late 1970s.^1,2^ Despite the remarkable success of cisplatin in cancer treatment, its use is limited by severe toxic side effects, inactivity against certain cancers, and tumor cell resistance. These drawbacks led scientists to search for other metal-based drugs, starting with the introduction of the cisplatin derivatives carboplatin and oxaliplatin,^3^ and then branching out to the utilization of nonplatinum metal compounds,^4–6^ especially those with biologically essential metals such as iron.^7–9^ Research on iron complexes as anticancer agents was encouraged by the promising anticancer properties of ferrocifens (ferrocenyl analogues of tamoxifen).^10^ Following the findings on ferrocifens, various monoiron­(II) half-sandwich complexes, such as [CpFe­(P–P)­(L)]^+^ (where Cp = η^5^-C_5_H_5_, P–P = bidentate phosphane, L = substituted imidazole (Imi-R) or nitrile (Nit) ligand), showed cytotoxicity against human cancer cell lines.^11–20^ In the case of iron­(II) cyclopentadienyl carbonyl complexes, such as [CpFe­(CO)­(PR_3_)­(L)]^+^ (L = Imi-R, Nit), [CpFe­(CO)­(P–P)]^+^, [CpFe­(CO)­(L)]^+^ (L = vinyl-aminoalkylidene ligand) and [CpFe­(CO)_2_X] (X = halide, SCN),^14–20^ the release of the carbon monoxide coligand could have a synergistic effect and lead to multitherapeutic actions.^19^ This expectation arises due to the numerous beneficial biological activities exhibited by CO, such as anti-inflammatory, antiproliferative, vasodilative, and neuroprotective effects.^21^ Several iron­(II) cyclopentadienyl carbonyl complexes have, in fact, been exclusively investigated as CO-releasing molecules (CORMs) owing to the potential of these systems to act as stimuli-responsive prodrugs for the controlled delivery of therapeutic amounts of CO to biological targets.^22–28^

The use of “bare” organometallic carbonyl complexes as CORMs may have some drawbacks, such as poor solubility of the parent complexes in aqueous media, low stability in aqueous media (resulting in uncontrolled CO-release kinetics), toxicity and/or precipitation of metal-containing decarbonylation fragments. One approach to mitigate these issues is to conjugate the CO-releasing moieties with macromolecular, inorganic or metal–organic carriers.^29–31^ Broadly speaking, these systems can be divided into conjugates with a covalently bound CORM-fragment, and conjugates with noncovalently encapsulated CORMs. There are a few examples of covalently bound Fe-CORM conjugates, e.g., Schmalz and co-workers described conjugates of oxycyclohexadiene-Fe­(CO)_3_ units with a plasmin-specifying peptide, itaconates and a lipoxin analogue,^32–34^ and Ou et al. anchored CpFe­(CO)_2_ units via pendant thiol groups to upconversion nanoparticles.^35^ To date, noncovalently encapsulated iron carbonyl complexes have not been specifically investigated as CORM conjugates. Among the potential carrier structures for noncovalent encapsulation of Fe-CORMs, cucurbit­[*n*]­urils (CB*n*) stand out as excellent candidates owing to their proven ability to form host–guest complexes with a range of organic and metal-based drugs,^36,37^ including anticancer platinum­(II),^38^ ruthenium­(II) arene,^39^ titanocene and molybdocene compounds.^40^ CB7, comprising 7 methylene-linked glycoluril units, has shown the greatest potential for use as a drug delivery vehicle because of its low toxicity *in vitro* and *in vivo*,^41^ relatively good solubility in water (compared with other CB*n* homologues),^40^ and ideal cavity size for the encapsulation of small-molecule drugs.^42–44^

CB7 forms inclusion complexes of high thermodynamic stability with ferrocene and substituted ferrocenes.^45–49^ The major driving force for the high-affinity binding of hydrophobic residues like the cyclopentadienyl group has been traced to the high enthalpic gain (possibly assisted by a smaller entropic contribution) that arises from the release of high-energy water molecules from the host cavity.^50,51^ The formation of inclusion compounds between CB*n* and half-sandwich complexes of iron does not appear to have been described at the time of writing. To address this situation, and to prepare new examples of noncovalently encapsulated Fe-CORMs, in the present work the iron complexes [CpFe­(CO)_2_Cl] (**1**) and [CpFe­(CO)_2_CH_2_CONH_2_] (**2**) have been encapsulated in CB7, yielding the respective inclusion compounds **1**@CB7 and **2**@CB7, which have been characterized in the solid-state by several techniques. CO release studies using the myoglobin assay show that the inclusion compounds are visible-light-activatable CORM-carrier conjugates.

## Results and Discussion

### Synthesis and Characterization of Inclusion Compounds

To isolate the inclusion compounds, complexes **1** and **2** were first dissolved in ethanol and then solid CB7 was added followed by water to promote dissolution of the host and formation of the inclusion complexes, which precipitated as pale orange (**1**@CB7) and pale yellow (**2**@CB7) solids (Scheme 1). CHN microanalyses and Fe determination by ICP-OES indicated that the initial Fe:CB7 molar ratio used in the syntheses was retained in the final products. To confirm successful inclusion complexation, **1**@CB7 and **2**@CB7 were characterized by ATR FT-IR, Raman and ^13^C­{^1^H} CP MAS NMR spectroscopies, PXRD and TGA.

**Scheme 1:**
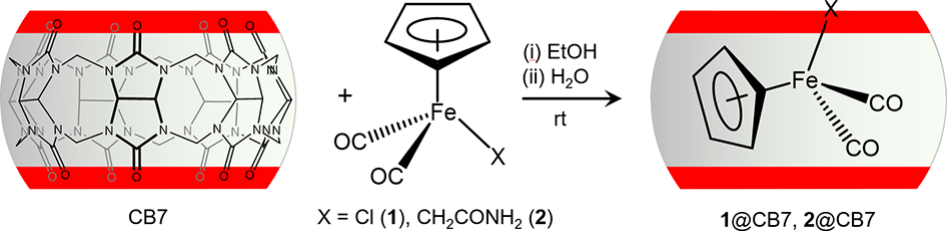
Synthesis of CB7 Inclusion Compounds

The ATR FT-IR and Raman spectra of **1**@CB7 and **2**@CB7 in the solid-state contain the characteristic bands of the macrocyclic host molecule, with no significant shifts being registered relative to pristine CB7 (Figure 1). Since CB7 does not exhibit bands in the range of 1900–2100 cm^–1^, the carbonyl (C≡O) stretching modes of the guest molecules are readily observed as bands at about 2007 and 2053 cm^–1^ for **1**@CB7, and 1965 and 2020 cm^–1^ for **2**@CB7, which are blue-shifted to varying degrees (10–30 cm^–1^) relative to those for the free complexes **1** and **2**. The spectral shifts, in addition to the narrowing of the IR band initially present at 1980 cm^–1^ for **1** and 1934 cm^–1^ for **2**, are consistent with noncovalent encapsulation which places the guest molecules in a solution-like isolated environment.^52^ A solution of complex **1** in carbon tetrachloride, for example, displays the IR ν­(CO) bands at 2012 and 2055 cm^–1^.^53^ For reference, the IR ν­(CO) frequencies for **1**@CB7 (2051, 2005 cm^–1^) are the same, to within 1 cm^–1^, as those reported previously for β-cyclodextrin and permethylated β-cyclodextrin inclusion compounds of **1**.^54,55^ The asymmetric broadening of the ν­(CO) bands below 2000 cm^–1^ for the free dicarbonyl complexes in the solid-state may be due to intermolecular interactions (CH···O and CH···π contacts, which are known to be present in carbonyl/Cp-containing organometallics^56^), which would not be present for the inclusion compounds.

**Figure 1:**
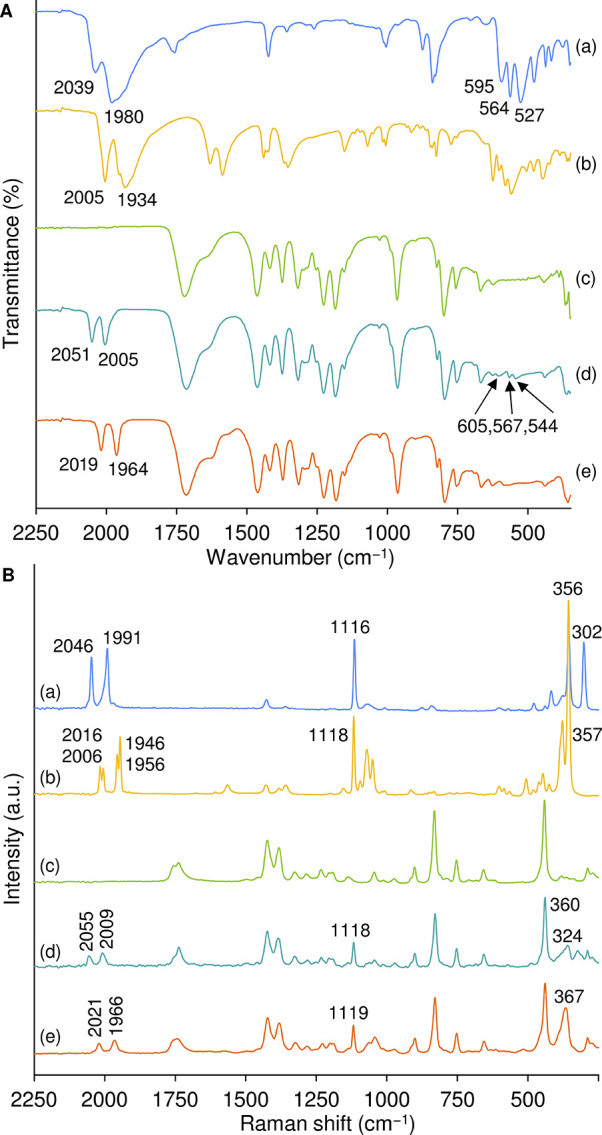
ATR FT-IR (A) and FT-Raman (B) spectra in the solid-state of (a) **1**, (b) **2**, (c) CB7, (d) **1**@CB7, and (e) **2**@CB7. The frequencies of selected bands are indicated.

Apart from the ν­(CO) bands, the other strong bands displayed by **1** in the Raman spectrum are those at 302, 356, and 1116 cm^–1^, which are assigned to ν­(Fe–Cl), ν­(Fe–Cp) and Cp ring breathing modes, respectively.^57,58^ Upon comparing the spectra for CB7 and **1**@CB7, bands at 324, 360, and 1118 cm^–1^ for **1**@CB7 can be attributed to these three modes of the encapsulated guest, with a significant shift only being observed for the Fe–Cl stretching mode. As expected, the latter band is absent for **2**@CB7 (and **2**), while bands at 367 and 1119 cm^–1^ are attributed to ν­(Fe–Cp) and Cp ring breathing modes of the encapsulated guest (cf. 357 and 1118 cm^–1^ for **2**). Strong bands at 595, 564, and 527 cm^–1^ in the ATR FT-IR spectrum of **1** are assigned to Fe–C–O bending modes.^53^ Since CB7 does not display bands in this region, the weak bands at 605, 567, and 544 cm^–1^ for **1**@CB7 are attributed to Fe–C–O bending modes of the encapsulated guest. The host-guest complex **2**@CB7 also displays weak bands in this region, but they overlap with each other and are not resolved.

Figure 2 shows the ^13^C­{^1^H} CP MAS NMR spectra of pristine CB7, **1**@CB7 and **2**@CB7, and the solution ^13^C­{^1^H} NMR spectra of the dicarbonyl complexes **1** and **2**. In accordance with the expected three-legged piano-stool geometry, the spectra of the free complexes show single resonances for the two CO ligands (211.7 ppm for **1**, 216.3 ppm for **2**) and for the Cp group (84.9 ppm for **1**, 86.1 ppm for **2**). The higher δ_CO_ and lower ν­(CO) for **2** are explained by the lower electron-withdrawing capacity of the CH_2_CONH_2_ ligand vs Cl. The resultant increase in electron density on the metal allows more back-donation to the CO ligands and hence more electron density in the π* orbitals of the C–O bonds, leading to a weaker C–O bond and lower ν­(CO).^27^ The higher carbonyl chemical shift for **2** is consistent with the known correlation (for transition metal carbonyl complexes) of increasingly deshielded carbonyl resonances with increasing metal → carbonyl π back-donation.^27,59,60^ Complex **2** shows two additional signals for the carbonyl (186.2 ppm) and CH_2_ (−1.5 ppm) moieties of the acetamide group. The solid-state NMR spectra of the inclusion compounds are dominated by the single peaks observed for the C=O, CH and CH_2_ groups of the macrocyclic host, which match those observed for the pristine CB7 sample. For **2**@CB7, additional resonances assigned to the guest include a relatively sharp signal for the Cp group at 86.3 ppm, and a very weak signal for the terminal CO ligands at 215.6 ppm, both of which are essentially unshifted relative to the corresponding resonances for **2** in solution. Slightly more intense signals for the acetamide group were present at 189.3 and –1.83 ppm. For **1**@CB7, peaks assigned to the carbonyl (ca. 216 ppm) and Cp (88.3 ppm) groups of the guest are shifted slightly downfield relative to those for **1** in solution.

**Figure 2:**
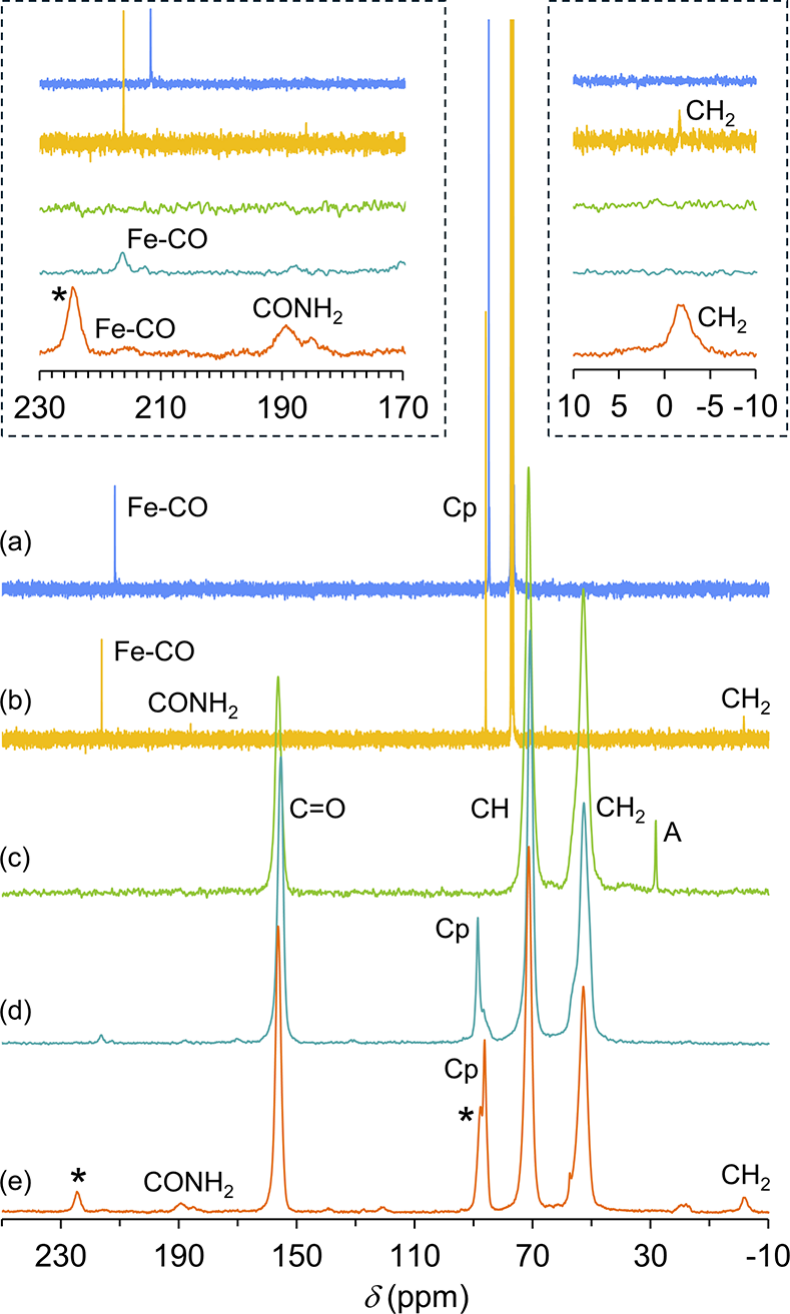
^13^C­{^1^H} CP MAS NMR spectra of (c) CB7, (d) **1**@CB7 and (e) **2**@CB7 compared with solution ^13^C­{^1^H} NMR spectra (CDCl_3_) of (a) **1** and (b) **2**. The insets show amplifications of the 170 to 230 ppm and –10 to 10 ppm regions. Asterisks denote spinning sidebands. For CB7 (c), the signal identified with A is due to acetone present in the as-prepared sample.

PXRD confirmed the amorphous nature of the as-synthesized CB7 sample (Figure 3c). It is noteworthy, however, that the trace matches with the intensity envelope observed in the PXRD pattern of a more crystalline CB7 sample reported previously,^48^ indicating that the two samples may have the same underlying structure (i.e., cucurbituril packing arrangement), albeit with very different degrees of long-range order. The host-guest complex **2**@CB7 also produced a halo pattern characteristic of amorphous solids (Figure 3e). This pattern is, nevertheless, slightly different from that for CB7 in that an additional broad reflection is present around 7.8° 2θ, possibly indicating a different packing arrangement for the host molecules and hence the presence of a genuine inclusion complex with **2**. For **1**@CB7, PXRD shows that the isolated solid was microcrystalline since several relatively sharp reflections are present in the range 5–25° 2θ (Figure 3d). The pattern is strongly indicative of inclusion complex formation because, first, it is distinct from that for as-prepared CB7 (Figure 3c), evidencing a change in structure (i.e., a change in the crystal packing arrangement of CB7 molecules) caused by the encapsulation of **1**, and, second, it does not contain reflections indicative of pure (nonincluded) **1** (Figure 3a).

**Figure 3:**
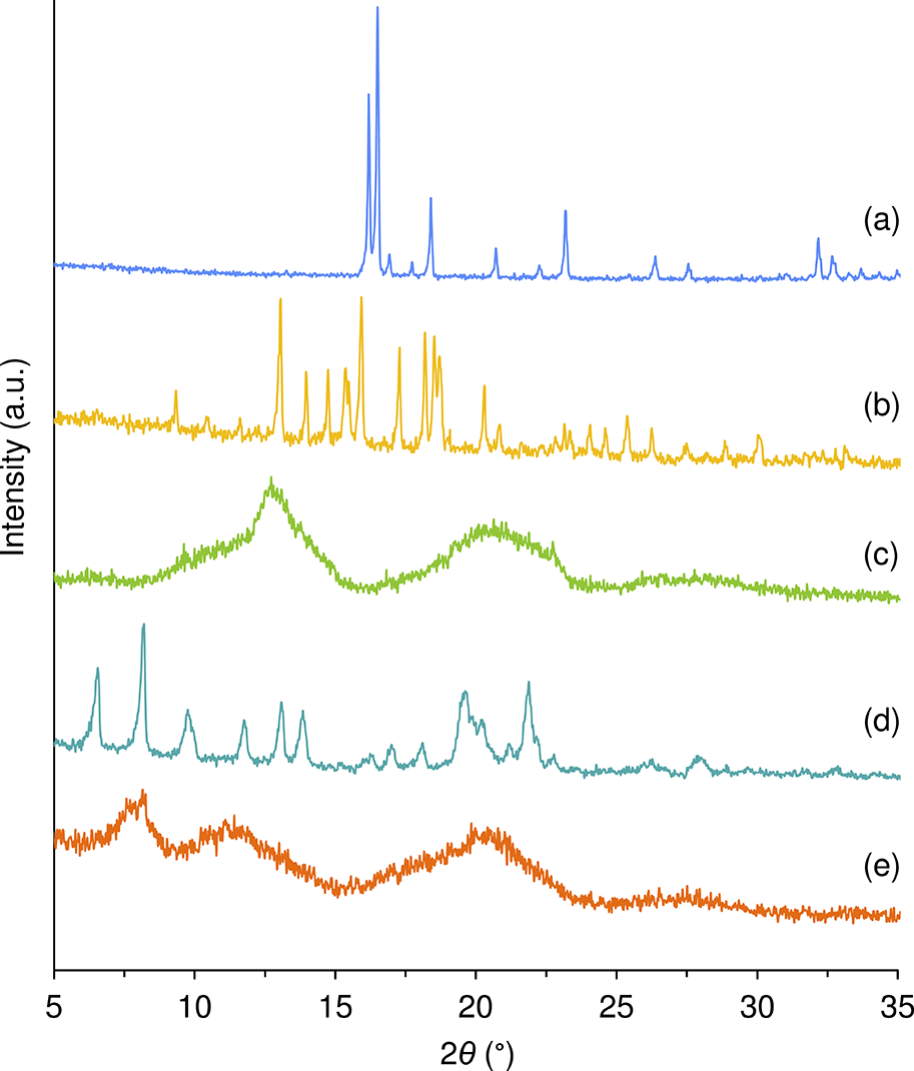
PXRD patterns of (a) **1**, (b) **2**, (c) CB7, (d) **1**@CB7, and (e) **2**@CB7.

Figure 4 shows the TGA profiles (recorded under air) of **1**, **2**, CB7, **1**@CB7 and **2**@CB7. The inclusion compounds exhibit a weight loss step from ambient temperature up to about 150 °C (11.6–14.6% mass loss), attributed to the loss of water. Following this step, both compounds display a single continuous mass loss across the temperature range of 250–400 °C, leaving residual masses of 5.5% for **1**@CB7 and 10.9% for **2**@CB7. This step is attributed to the overlapping oxidative decomposition of the organometallic guest and the CB host. When compared with the TGA of CB7, the decomposition of the inclusion compounds is shifted to lower temperature by 45–60 °C, which may be due to the promoting effect of organometallic degradation on the decomposition of the macrocyclic host. This hypothesis is further supported by comparing the TGA curves of the inclusion compounds with those of complexes **1** and **2**. In the temperature range of 90–200 °C, the free complexes show significant weight loss steps that arise from thermally promoted decarbonylation/sublimation/decomposition: two sequential steps for **1**, with an onset temperature of 95 °C, leading to a mass loss of 33.1% at 195 °C, and a single step for **2**, with an onset temperature of 140 °C, leading to a mass loss of 35.5% at 175 °C. The TGA curves of the inclusion compounds do not display any resolved steps between 90 and 200 °C that could be associated with these thermal events of **1** or **2** (either nonincluded or encapsulated). Thus, thermal events such as decarbonylation of **1** and **2** are inhibited by full encapsulation by the CB7 host, with the oxidative decomposition of the organometallic guest taking place concurrently with CB decomposition above 250 °C.

**Figure 4:**
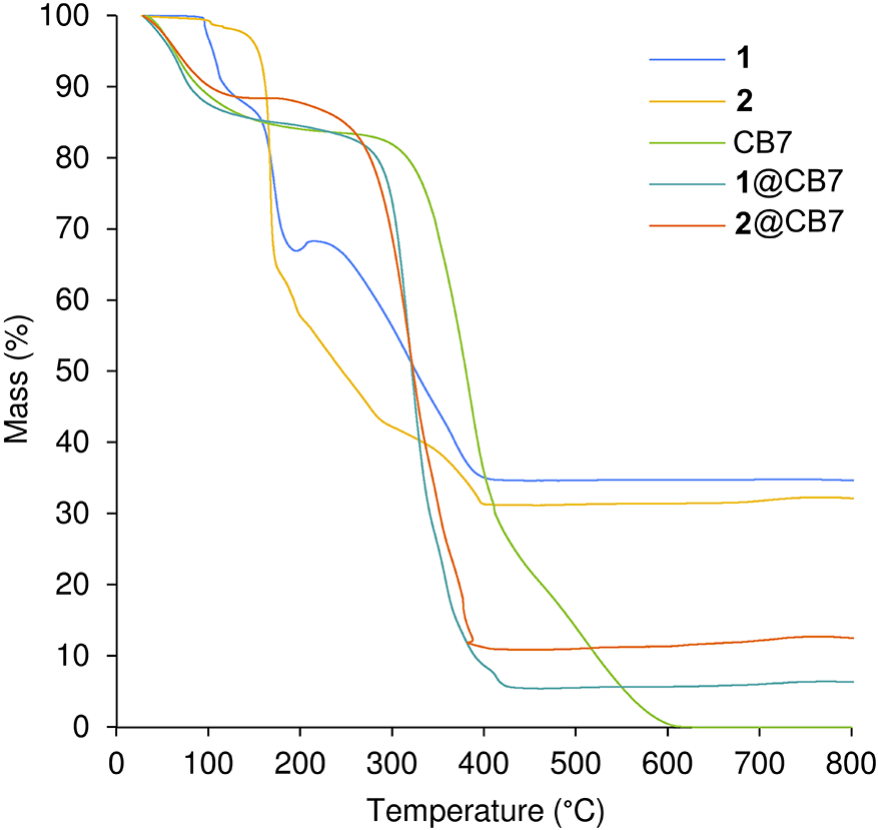
TGA curves (measured under air) of **1**, **2**, CB7, **1**@CB7, and **2**@CB7.

To check the stabilities of the synthesized compounds, solid samples of **1**, **2**, **1**@CB7 and **2**@CB7 were exposed to ambient light and air for 4 weeks, and ATR FT-IR spectra were recorded every 7 days. No measurable spectral or color changes were observed during this period, indicating that the free complexes and inclusion compounds had high stability in the solid-state (Figures S1 and S2 in the Supporting Information).

### Single-Crystal X-ray Structure Analysis

Single crystals of **2**@CB7 suitable for X-ray diffraction were obtained after slow evaporation of the mother liquor, separated during the synthesis procedure, in the dark and under air. The compound crystallizes in the monoclinic centrosymmetric space group *P*2_1_/n with the asymmetric unit being composed of two crystallographically independent [**2**@CB7] binary complexes that differ only in the orientation of the [CpFe­(CO)_2_CH_2_CONH_2_] molecules inside the CB host cavity (Figure 5). The asymmetric unit is further composed of a total of 40.65 highly disordered water molecules of crystallization and 0.7 ethanol molecules of crystallization.

**Figure 5:**
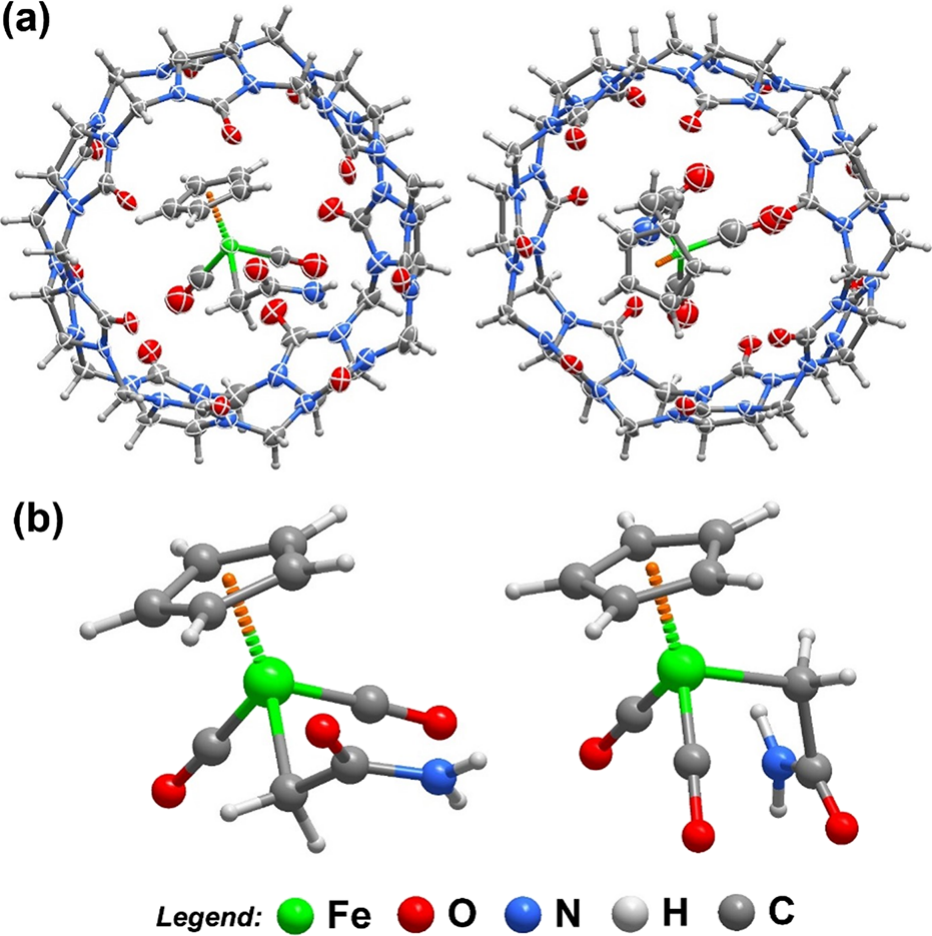
(a) Schematic representation of the two crystallographically independent [**2**@CB7] complexes found in the crystal structure of **2**@CB7. Non-hydrogen atoms corresponding to the asymmetric unit are depicted as ellipsoids drawn at the 50% probability level and hydrogen atoms are drawn as small spheres with arbitrary radius (water molecules of crystallization have been omitted for clarity). (b) Representation of the two independent [CpFe­(CO)_2_CH_2_CONH_2_] molecules present inside the CB7 cavities, showing the rotation of the pendant amide group.

In the two crystallographically independent [**2**@CB7] binary complexes, the guest molecules [CpFe­(CO)_2_CH_2_CONH_2_] are intact, being composed of a cyclopentadienyl ring, two carbonyl groups and a pendant acetamide group coordinated to the iron­(II) metal center with Fe–C bond distances ranging between 2.035(12) and 2.091(11) Å for Fe1, and between 2.053(11) and 2.116(12) Å for Fe2. The position of the iron metal center regarding the geometric center of the host CB7 is, however, different, with distances of 0.59 Å for Fe1 and 1.46 Å for Fe2. This difference in conformation inside the CB7 host is more evident in the angle formed by the Cp ring and the CB7 axis (intersecting the centers of the two cavities), with angles of 84.5° for Fe1 and 3.5° for Fe2. In fact, the Fe2 [CpFe­(CO)_2_CH_2_CONH_2_] complex has the Cp ring outside of the CB7 cavity as depicted in Figure 6. These results show that the dicarbonyl complex has a reasonable degree of rotational freedom inside the CB7 cavity, mostly because it is solely maintained there by very weak C–H···O hydrogen bonding interactions (not shown).

**Figure 6:**
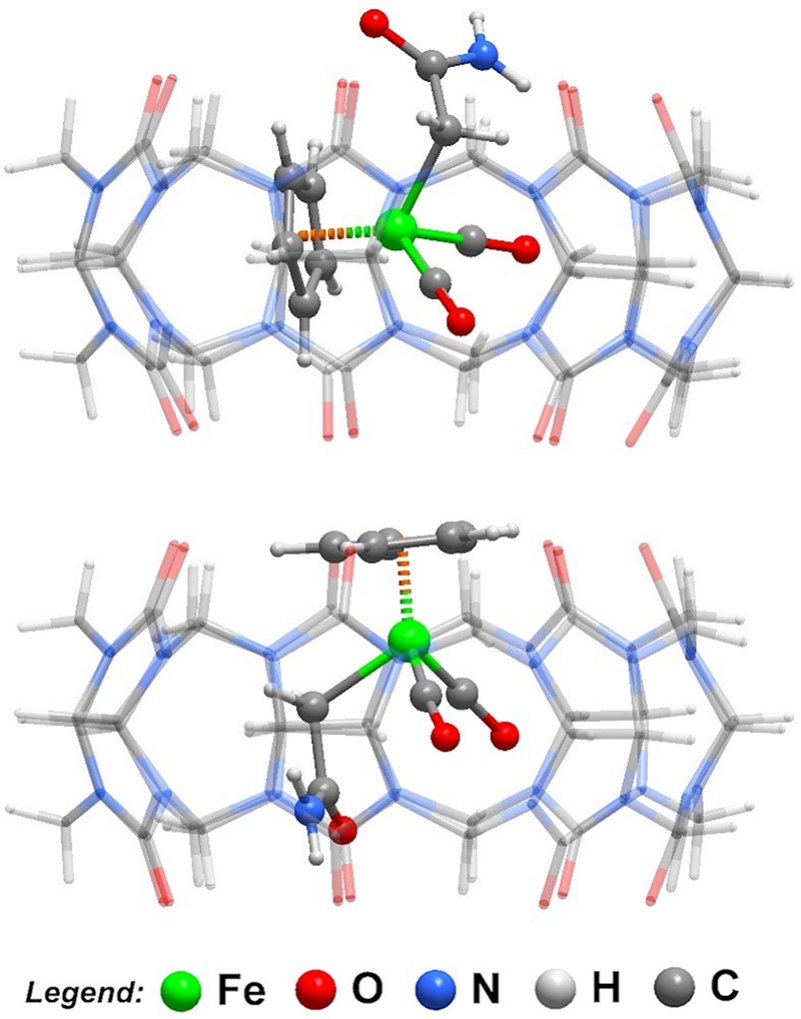
Lateral view of the two crystallographically independent [**2**@CB7] complexes found in the crystal structure of **2**@CB7.

### Host–Guest Complexes in Aqueous Solution

Electrospray mass spectrometry (ESI-MS) has been proven to be a powerful tool to analyze the formation of CB-based host–guest complexes in aqueous solution.^61^ The studies involving organometallic (cyclopentadienyl) guests have so far been limited to ferrocenylguanidinium and [CpMo­(CO)_3_Me] derivatives.^62,63^ In this work, the formation of complexes in aqueous phase was evaluated by ESI high resolution MS (HRMS). Free **1** and **2** guests were studied first. The infusion of an aqueous solution of **1** (100 μM) revealed the formation of several signals together with the sodiated ion (*m*/*z* 234.922) expected from its structure under positive polarity (Figure S3 in the Supporting Information). As the presence of water and the spray conditions can promote the transformation of **1**, an ethanol solution (100 μM) was used to test the formation of stable ions under a wide range of spray and ion optics conditions. Figure S4 presents the spectrum of a 100 μM solution of **1** in ethanol using a heater temperature of 50 °C and a capillary temperature of 70 °C. This low capillary temperature was essential to obtain stable ions of **1**. The fragmentation of the observed signal leads to release of the carbonyl groups, in agreement with the assignment (Figure S5). However, in water, even under these soft ionization conditions, signals other than the ion of intact **1** were observed, indicating its transformation in aqueous phase and/or during the spray. On the other hand, a clear signal of free **2** can be readily observed from aqueous solutions (Figure S6) that upon chemical induced dissociation (CID) also releases the carbonyl groups (Figure S7).

**1**@CB7 aqueous complexes revealed a set of gas phase signals compatible with chemical species other than the **1**@CB7 complex (Figure S8), which may be connected with the observed instability of **1** in aqueous phase and/or during the spray. Therefore, the formation of complexes between **1** and CB7 in aqueous solution was not clear from our ESI-HRMS data. Conversely, **2**@CB7 complexes are readily detected after the spray of a 1:1 stoichiometry of **2** and CB7 at 100 μM concentration (Figure 7) confirming their formation and stability in aqueous solution. The fragmentation of *m*/*z* 699.675 under higher energy collisional dissociation (HCD) confirmed the assignment (Figure S9).

**Figure 7:**
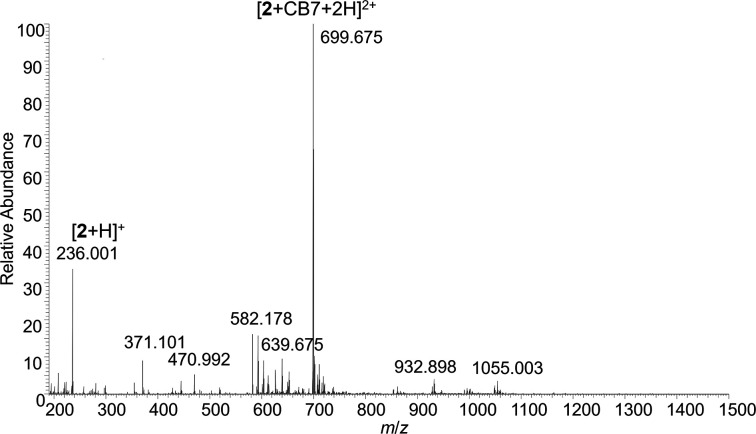
ESI-HRMS full scan spectrum of **2**@CB7 in aqueous solution (positive ESI).

To obtain additional evidence for the formation of **2**@CB7 host–guest complexes, we carried out a ^1^H NMR investigation of the binding interaction between **2** and CB7 in D_2_O. A preliminary NMR spectrum was obtained for a solution containing equal concentrations of **2** and CB7 (1 mM, Figure S10 in the Supporting Information). This experiment showed that the signals of free guest **2**, observed at 1.45 (CH_2_) and 4.83 ppm (Cp), shifted upfield and broadened considerably upon addition of 1 equiv of CB7. Since the upfield-shifted Cp signal overlapped with the HOD/H_2_O signals, further NMR spectra were recorded using a pulse sequence for water suppression to allow a clearer picture of the evolution of the signals of complex **2** (Figure S11). Specifically, spectra were recorded for the free **2** (2 mM) and for solutions prepared by the addition of different amounts of CB7 to give host:guest molar ratios of 0.25:1, 0.5:1, and 1:1. These experiments confirmed the preliminary results, i.e., interaction between **2** and CB7, as indicated by the upfield shift and broadening of the signals of encapsulated **2**, with considerable changes taking place upon addition of 1 equiv of CB7. The upfield shifts are attributed to the shielding effect exerted by the hydrophobic cavity of CB7, i.e., the data indicate that a 1:1 inclusion complex is formed in which the Cp and CH_2_ groups of the guest are located inside the macrocycle. No changes in the ^1^H NMR signals of encapsulated **2** (1:1 complex, D_2_O, 4 mM) were observed after 1 week, confirming the stability of the inclusion complexes in solution. The broadening of the signals due to encapsulated **2** is attributed to fast chemical exchange on the NMR time scale.

### UV–vis Stability Studies

To assess the stability of complexes **1** and **2** under simulated physiological conditions, degassed 100 μM solutions in 10 mM phosphate buffered saline (PBS, pH 7.4) were prepared and kept either in the dark at 37 °C (for **1**) or (for **1** and **2**) under constant exposure to cold white light (*E* = 10 mW cm^–2^) at 37 °C. UV–vis absorption spectra were collected over a period of 2–3 h (Figures S12–S14 in the Supporting Information). No significant spectral changes were observed for the solution of **1** kept in the dark (Figure S12), indicating that the complex is relatively stable under these conditions. The main absorption bands are found with maxima at 283, 341, and 392 nm, with an additional very weak and broad absorption in the visible region centered at 488 nm (Figure S15). For comparison, the absorption spectrum of **2** in 10 mM PBS displays bands at 260 and 360 nm, with the latter having a very weak low energy shoulder. Low energy bands (λ_max_ = 300–400 nm) in the electronic absorption spectra of [CpFe­(CO)_2_X] complexes, where X = Cl, I, or Br, have generally been assigned to ligand field transitions.^64–66^ The bands at 341 nm for **1** and 360 nm for **2** tail into the visible region (>400 nm, giving rise to the yellow/orange colors), prompting the use of blue light to photolyze the complexes. It was indeed found that irradiation of **1** and **2** in PBS solution with a cold white LED floodlight led to immediate UV–vis spectral changes (Figures S13 and S14). For **1**, irradiation resulted in an abrupt decrease in the low energy band at 392 nm, and an apparent transformation of the weak band at 341 nm to a shoulder with λ_max_ > 350 nm. Concurrently, the spectra recorded between 3 and 15 min show the growth of a high energy shoulder at ca. 230 nm, and the intense band at 283 nm transformed to a broad unresolved shoulder. For the spectra recorded between 30 and 120 min, an isosbestic point was observed at 262 nm, indicating that over this period the reaction was uncomplicated by side or subsequent reactions. Upon irradiation of the solution of **2** in PBS, the bands initially at 260 and 360 nm decreased such that only broad shoulders were observed in the spectrum recorded at 15 min. With longer irradiation times, the spectral sequence showed a growth in the lowest energy shoulder (to become a resolved peak with λ_max_ = 358 nm), and a further slight decrease in the highest energy shoulder, albeit accompanied by a more distinguishable peak maximum.

Photochemical investigations of the complex [CpFe­(CO)_2_Cl] (**1**) under liquid-phase conditions (with organic solvents) and in low temperature matrices have shown that after excitation either the halogen ligand Cl is split off (through heterolytic cleavage of the Fe–Cl bond, leading to the dimer [CpFe­(CO)_2_]_2_ as the principal product) or, more predominantly, the Fe–CO bond is broken, leading to dissociative loss or exchange of one CO ligand.^64,65,67–69^ Under liquid-phase conditions, the solvent plays a noninnocent role, with irradiation at λ > 400 nm showing no photochemical reaction in nonpolar solvents such as cyclohexane and benzene, and some polar aprotic solvents such as acetonitrile and THF.^67,68^ Jiang et al. used IR and ^1^H NMR spectroscopies to study photoinduced CO release from **1** in DMSO, D_2_O, and physiological saline/D_2_O (NaCl, 0.15 M).^24^ In DMSO, the photodecomposition of **1**, shown by the decrease of the ν­(CO) bands, was much faster with blue-light irradiation than with green- or red-light irradiation. The higher stability of the dimer [CpFe­(CO)_2_]_2_ under blue-light irradiation indicated that it was not a major product in the photodecomposition of **1**. ^1^H NMR studies in *d*_6_-DMSO showed that free cyclopentadiene was formed during the irradiation of **1**. With D_2_O or physiological saline as the solvent, IR spectroscopy evidenced both the direct photodecarbonylation of **1** and the formation of an intermediate, which was proposed to be a Cp-free dicarbonyl species, {Fe^II^(*cis*-CO)_2_}, with coordination around the metal center being satisfied by D_2_O and/or Cl^–^. The authors concluded that the photoinduced CO release in aqueous media was D_2_O-assisted. One aspect not addressed by this study was the quantification of CO released from **1**.

UV–vis stability studies were also conducted for **1**@CB7 and **2**@CB7 (Figures S16–S19 in the Supporting Information). In general, the UV–vis spectra of freshly prepared solutions of the inclusion compounds in 10 mM PBS showed bands (either as shoulders or clear peaks) that could be readily attributed to the encapsulated guest molecules (Figures S18 and S19). No appreciable shifts in band position were observed when compared with the spectra of free **1** and **2**. This was particularly evident for **2**@CB7 and the bands with maxima at 260 and 359 nm that match those observed for **2** (Figure S19). Upon irradiation of the solutions containing the inclusion compounds with cold white light, the resultant spectral changes generally paralleled those observed for **1** and **2**, although the new bands with λ_max_ > 350 nm appeared to a lesser extent (Figures S16–S19).

### CO Release Studies

The myoglobin (Mb) assay is the standard method employed for determining the amount and rate of CO released from CORMs.^70,71^ In this assay, the CORM is added to a solution of deoxy-Mb and the formation of carbonmonoxymyoglobin (MbCO) is followed spectrophotometrically through changes in either the Q-band (540–580 nm) or Soret band (400–450 nm) regions of the heme group. In the present work, CO release from 20 μM solutions of **1**, **2**, **1**@CB7 and **2**@CB7 was determined by analysis of changes in the Q-band region of the UV–vis spectra of Mb (38–47 μM) in phosphate buffer at physiological pH (7.4) and temperature (37 °C). The pristine host, CB7, was chosen as a negative control. As expected, CB7 did not cause any change in the spectrum of deoxy-Mb, confirming that the concentration of deoxy-Mb remained constant.

The CO-release data for the free complexes are collected in Table 1 and compared with data reported for other cyclopentadienyl iron­(II), molybdenum­(II) and tungsten­(II) carbonyl CORMs (also determined by the Mb assay). To aid comparison of CO-release rates, half-lives, *t*_1/2_, have been calculated, and are defined as the time taken for a CORM with a concentration of X μM (20 μM in the present work) to produce an MbCO concentration of X/2 μM (10 μM in the present work). Since the amounts of CO released do not always reach 0.5 equiv over the course of the assay, quarter-lives, *t*_1/4_ (time to reach [MbCO] = X/4 μM) are also presented to allow all compounds to be compared.

**Table 1:** CO Release Data for 1 and 2 Compared with Literature Data for Half-Sandwich Complexes^a^

compound	medium^b^	[CORM] (μM)	[Mb] (μM)	*T* (°C)	irradiation conditions	CO release^c^ (mol_CO_ mol_M_^–1^)	*t*_1/4_ ^d^ (min)	*t*_1/2_ ^d^ (min)	ref.
**1**	PBS	20	38–47	37	LED, 400-700 nm, 10 mW cm^–2^	1.16 ± 0.05 (45)	2.9 ± 0.6	6.8 ± 0.7	This work
					in the dark	0.27 ± 0.02 (120)	104 ± 14	-	This work
**2**	PBS	20	38–47	37	LED, 400-700 nm, 10 mW cm^–2^	1.38 ± 0.07 (135)^e^	8.9 ± 0.7	19.2 ± 1.9	This work
[(η^5^-C_5_H_4_CO_2_Me)-Fe(CO)_2_Br]	PB	40	66	37	no irradiation	0.68 (60)	15.5	38	^22^
[(η^5^-C_5_H_4_CO_2_Me)-Fe(CO)_2_Cl]	PB	40	66	37	no irradiation	nr^f^	-	63	^22^
**1**	PB	40	66	37	no irradiation	nr	-	350	^22^
[CpFe(CO)_3_]Cl	PB	40	66	37	no irradiation	nr	-	69	^22^
[CpFe(CO)_2_(η^1^-*N*-maleimidato)]	PBS/EtOH	67	33.5	rt	Halogen 330 lm, 450–650 nm	0.43 (140)^e^	54	-	^25^
[CpFe(CO)_2_(η^1^-*N*-succinimide)]	PBS/EtOH	67	33.5	rt	Halogen 330 lm, 450–650 nm	0.43 (125)^e^	50	-	^25^
[CpW(CO)_2_(η^1^-*N*-maleimidato)]	PBS/EtOH	100	33.5	rt	Halogen 330 lm, 450–650 nm	0.27 (135)^e^	106	-	^25^
[CpMo(CO)_2_(η^1^-*N*-maleimidato)]	PBS/EtOH	67	33.5	rt	Halogen 330 lm, 450–650 nm	0.43 (120)^e^	50	-	^25^
[CpMo(CO)_3_Me]	PBS/DMSO	20	45	37	in the dark	1.49 (120)^g^	12	25	^63,72^
	PBS/DMSO	20	40	37	in the dark	0.06 (30)^h^	-	-	^63^
	PBS/DMSO	20	40	37	UV (365 nm), 2.5 mW cm^–2^	1.17 (30)^h^	∼3	6.3	^63^
[(η^5^-C_5_H_4_CO_2_Me)-Mo(CO)_3_Me]	PBS/DMSO	20	40	37	UV (365 nm), 2.5 mW cm^–2^	0.88 (360)	∼7.5	85	^72^
					in the dark	0.54 (360)	84	325	^72^
[CpMo(CO)_3_Cl]	PBS	20	60	37	no irradiation	1.3 (33)	10	14.4	^73^
[CpMo(CO)_3_(C≡CR)]^i^	PBS	-	60	37	no irradiation	0	-	-	^73^
[CpMo(CO)_3_(C≡CR)]^j^	PBS	60	60	37	no irradiation	0.037 (120)	-	-	^73^
		60	60	37	UV (325 nm), 6W	0.75 (30)	10	16	^73^
		20	60	37	UV (325 nm), 6W	1.9 (55)	8.3	13	^73^
[CpMo(CO)_3_(L^1^)](BF_4_)^k^	PBS/DMSO	40	50	37	no irradiation	0.18 (60)	-	-	^23^
[CpMo(CO)_3_(L^2^)](BF_4_)^k^	PBS/DMSO	40	50	37	no irradiation	1.22 (60)	6.1	11.3	^23^
[CpFe(CO)_2_(L^1^)](BF_4_)^k^	PBS/DMSO	60	50	37	no irradiation	0 (60)	-	-	^23^
[CpFe(CO)_2_(L^2^)](BF_4_)^k^	PBS/DMSO	60	50	37	no irradiation	0 (60)	-	-	^23^

a CO release determined by myoglobin assays.

b PBS = phosphate buffered saline (pH 7.4), PB = phosphate buffer (pH 6.8).

c Time in minutes in parentheses.

d Defined as the time taken for a CORM with a concentration of X μM to produce an MbCO concentration of X/2 μM (*t*_1/2_) or X/4 μM (*t*_1/4_).

e No MbCO formation for assays in the dark.

f nr = not reported.

g CO release was facilitated by the reducing agent sodium dithionite used in the Mb assay (0.4%).

h Dithionite interference minimized by using 0.1% in the Mb assay. When 0.2% dithionite was used instead of 0.1%, the amount of CO released after UV irradiation for 30 min was 1.43 mol_CO_ mol_Mo_^–1^, with no change in *t*_1/2_.

i R = Ph or CH_2_OCH_2_Ph. These complexes were insoluble in the aqueous medium used for the Mb assay, and no CO release was subsequently detected.

j R = CH_2_O-β-D-fructopyranose.

k L = η^1^-2-pyrone = η^1^-{O}-C­{=O}-O-CMe=CH-COMe=CX (X = H (L^1^), Br (L^2^)).

The CO release behavior of **1** was first reported by Scapens et al.^22^ They found that **1** was a very slow releaser (in the absence of photoirradiation^74^) with *t*_1/2_ = 350 min. In the present work, *t*_1/2_ could not be determined for the 2 h assay performed with **1** in the dark (Figure 8), but the *t*_1/4_ value of 104 ± 14 min is consistent with the result reported by Scapens et al. After 2 h, **1** released 0.27 ± 0.02 equiv of CO, with no plateau reached. This result is somewhat at odds with the UV–vis studies which indicated no reaction over 2 h for a solution of **1** in PBS kept in the dark (Figure S12). It is possible that the decarbonylation of **1** was too small for spectral changes to become apparent. In the report by Scapens et al., *t*_1/2_ values for CO loss ranged from 38 min for [(η^5^-C_5_H_4_CO_2_Me)­Fe­(CO)_2_Br] to 350 min for **1**, with ten other cyclopentadienyl iron carbonyls displaying intermediate values (Table 1).^22^

**Figure 8:**
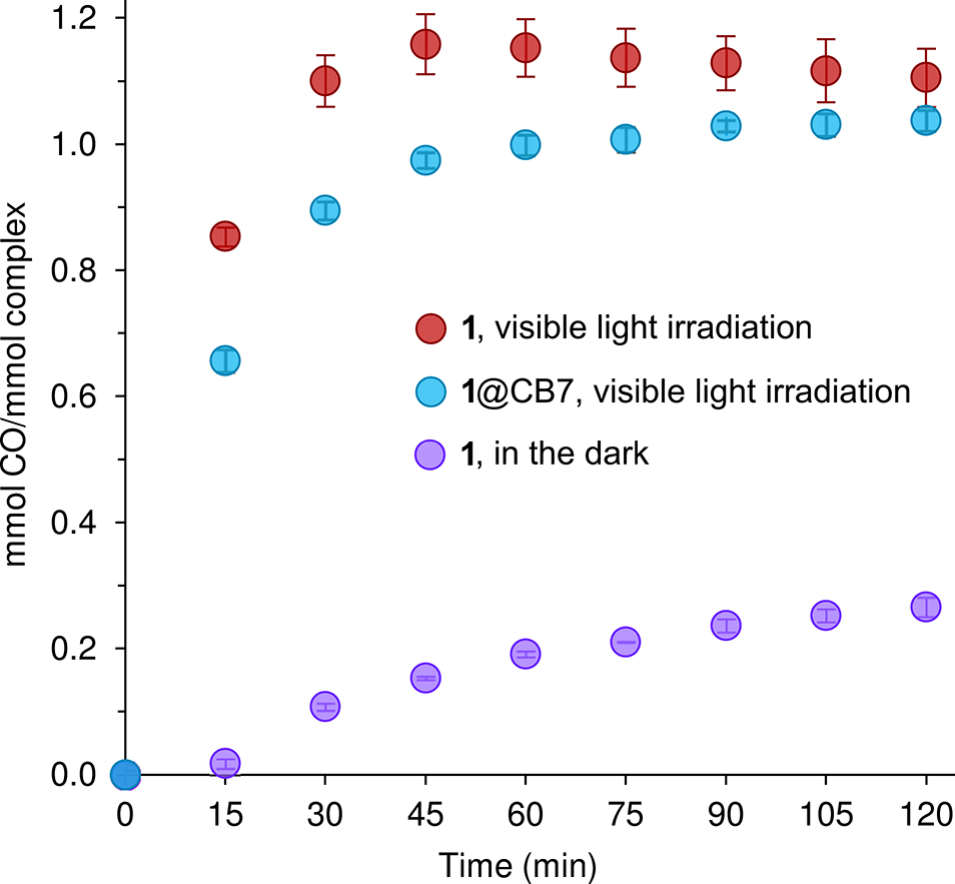
Time courses of CO release (Mb assay, 37 °C, pH 7.4) for complex **1** and **1**@CB7 under visible light irradiation (λ > 400 nm), and **1** in the dark. The data values are the mean ± standard deviation of three independent assays.

It is noteworthy that complex **2** showed no loss of CO for the Mb assay carried out in the dark (Figure 9), in agreement with the knowledge that the complex is rather air stable and does not release CO in normoxic aqueous media for at least 6 h.^75^ Hence, at physiological pH, the Fe-alkyl bond in **2** does not undergo hydrolysis, whereas the Fe–Cl bond in **1** is likely to undergo such a reaction which initiates decomposition and CO release, albeit at a slow rate.

**Figure 9:**
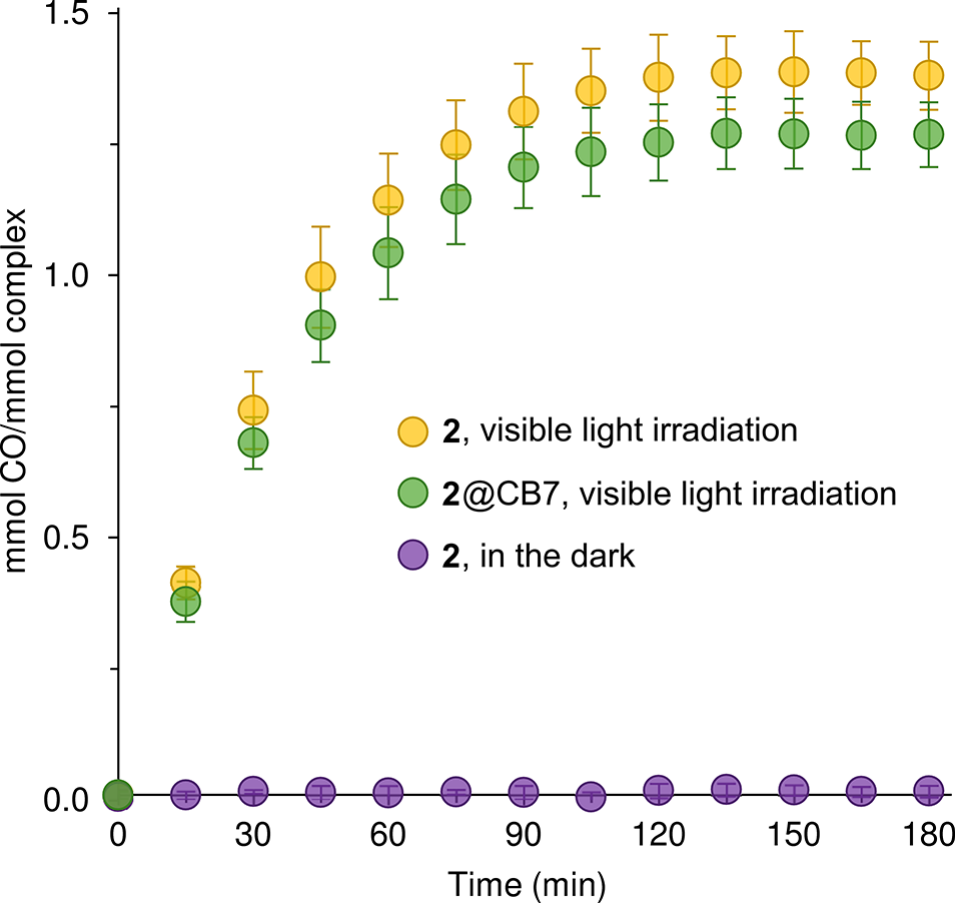
Time courses of CO release (Mb assay, 37 °C, pH 7.4) for complex **2** and **2**@CB7 under visible light irradiation (λ > 400 nm), and **2** in the dark. The data values are the mean ± standard deviation of three independent assays.

High dark stability was also reported for [CpM­(CO)_2_(η^1^-*N*-imidato)] complexes (M = Fe, Mo, W).^25^ On the other hand, several Mo­(II) tricarbonyl complexes have been reported to release significant amounts of CO during Mb assays performed in the dark. Particularly, the complexes [CpMo­(CO)_3_X] (X = Cl, Me) and [CpMo­(CO)_3_(η^1^-2-pyrone)]­(BF_4_) released between 1.2 and 1.5 mmol of CO per mmol of complex after 0.5–2 h, with half-lives between 11 and 25 min (Table 1).^23,63,72,73^ Tests revealed that CO release from [CpMo­(CO)_3_Me] (in the dark) was promoted in a concentration-dependent manner by the reducing agent sodium dithionite used in the Mb assay.^63^ This behavior mimics that found for two well-known CORMs, [Ru­(CO)_3_Cl_2_]_2_ (CORM-2) and [Ru­(CO)_3_Cl­(glycinate)] (CORM-3), which were originally characterized as fast CO releasers based on the Mb assay, but were later found to be very slow releasers in the absence of dithionite.^76,77^ It is presently unclear if the significant dark-release observed for the aforementioned Fe­(II) and Mo­(II) complexes is promoted in any way by interaction of the complexes with dithionite.

The Mb assays with **1** and **2** were subsequently performed with photoirradiation using a cold white LED floodlight (*E* = 10 mW cm^–2^). CO release was promoted by irradiation, with one mmol of CO per mmol of complex being released fairly rapidly (20 min for **1**, 45 min for **2**), followed by a plateau starting at 45 min for **1** (1.16 ± 0.05 equiv of CO) and 135 min for **2** (1.38 ± 0.07 equiv of CO) (Figures 8 and 9). The faster CO release from **1** is reflected in the higher initial rate (1.13 μM min^–1^ vs 0.54 μM min^–1^ for **2**, calculated at 15 min) and the lower *t*_1/2_ (6.8 ± 0.7 min vs 19.2 ± 1.9 min for **2**). The apparent higher lability of CO in **1** may be partly linked with a weaker Fe-CO bond (which, as discussed above, is indicated by the comparison of the carbonyl ^13^C NMR chemical shifts and IR stretching frequencies).^22,27^ To the best of our knowledge, the only monoiron cyclopentadienyl carbonyls that have previously been established as photochemically activated CORMs are [CpFe­(CO)_2_(η^1^-*N*-imidato)][Bibr ref25] and chalcogenopropargylcarbonato complexes.^78^ However, CO release was only quantified (via the Mb assay) for the η^1^-*N*-imidato complexes. A comparison of the CO release data for these complexes with those for **1** and **2** is of limited value because the imidato complexes were studied with a lower assay temperature (rt), different light source (halogen lamp), and an excess of complex (67 μM) vs Mb (33.5 μM). Based on the reported CO release curves, the imidato complexes were able to convert deoxy-Mb to MbCO with *t*_1/4_ = 50–54 min, while under the conditions used in the present work the *t*_1/4_ values were 2.9 ± 0.6 min for **1** and 8.9 ± 0.7 min for **2**.

When compared with other half-sandwich CORMs (Table 1), complexes **2** and, especially, **1**, are relatively fast CO releasers, despite the use of a low-power visible light source (*E* = 10 mW cm^–2^) for photoactivation. This could make the complexes suitable for a wide range of applications since a key feature of photoactivatable CORMs (photoCORMs) is that varying the light intensity may allow precise dosage control.^79^ Complex **2** has the added advantage of being stable in the dark. Considering the amounts of CO released and the half-lives, the visible light-activated decarbonylation profiles of **1** and **2** are similar to those found for the UV (325–365 nm) light-induced decarbonylation of [CpMo­(CO)_3_Me][Bibr ref63] and [CpMo­(CO)_3_(C≡CR)] (R = CH_2_O-β-D-fructopyranose),^73^ respectively (Table 1). In photoCORM research, one of the main thrusts has been to shift the photoactivation from the UV region to longer wavelengths to minimize photodamage to healthy cells and tissues and to achieve deeper tissue penetration.^80,81^ Among visible light photoCORMs, manganese­(I) tri- and tetracarbonyl complexes have featured prominently.^82^ The CO release behaviors of **1** and **2** are comparable with those typically observed for Mn­(I) photoCORMs when irradiated with blue light.^82^ For example, photoirradiation of phenanthroline-based complexes of the type *fac*-[MnX­(CO)_3_(L)] (X = Br, N_3_) with blue (435–450 nm) LED light resulted in the release of ca. 1.4 mmol of CO per mmol of complex, with half-lives ranging between 6 and 24 min.^83^

Figures 8 and 9 show the CO release profiles obtained for **1**@CB7 and **2**@CB7 with photoirradiation. For **2** and **2**@CB7, the amounts of CO released (at 135 min) and the half-lives are the same within the margin of error, while for **1** and **1**@CB7 there was a slight increase in *t*_1/2_ from 6.8 ± 0.7 min to 10.0 ± 0.6 min, and an approximate 10% reduction in the amount of CO released. As mentioned above, complex **1** is a slow CO releaser when incubated in the dark under simulated physiological conditions. The processes responsible for CO labilization in the dark, namely hydrolysis and ligand exchange, are likely to be hampered for the complex encapsulated in the hydrophobic cavity of CB7, which could explain the slightly slower CO release observed for **1**@CB7 (with photoirradiation) in comparison to the free CORM. For **2**@CB7, the full retention of the photoactivatable properties observed for **2** in solution may be largely because of the aforementioned “solution-like” environment experienced by the guest molecule in the inclusion complex.

In Table 2, the CO release results (Mb assay conditions, CO release amounts, half/quarter-lives) for **1**@CB7 and **2**@CB7 are compared with data obtained by the Mb assay for previously reported noncovalently encapsulated CORMs (apart from entry 8 which details a polymethylstyrene derivatized with Cp_2_Fe_2_(CO)_4_@β-cyclodextrin inclusion complexes). For the literature data, the hosts include organic carriers (cucurbiturils and β-cyclodextrin (β-CD) (entries 3–7), polylactide fibers (entry 9), and silk fibroin nanoparticles (SFNs; entry 11)), inorganic carriers (layered double hydroxide (LDH; entry 13), ordered mesoporous silicas (entries 17–19)), and metal–organic frameworks (MOFs; entries 14, 16, 22, 24). Where available (for the literature results), data for the free CORMs have been included (if not already listed in Table 1). For the systems closest to **1**@CB7 and **2**@CB7, i.e., entries 3–7, encapsulation of the complexes [(η^5^-C_5_H_4_R)­Mo­(CO)_3_Me] (R = H, CO_2_Me) in CB7, CB8 or β-cyclodextrin had the effect of slowing down the rate of CO release for assays performed with UV light (365 nm) irradiation.^63,72^ The results for [CpMo­(CO)_3_Me]@CB7 (*t*_1/2_ = 10 min vs 6.3 min for the free complex) are very similar to those for the visible light-induced CO release from **1**@CB7 (*t*_1/2_ = 10.0 ± 0.6 min vs 6.8 ± 0.7 min for the free complex). For the encapsulation of [CpMo­(CO)_3_Me] in β-CD (entry 4), the resultant *t*_1/2_ of 13.2 min is slightly higher than that for the corresponding CB7 complex, but only 0.7 equiv of CO were released after 30 min, i.e., about half the number released from the free complex (the assay duration was restricted to 30 min due to the lower amount of dithionite used).^63^ Encapsulation of [(η^5^-C_5_H_4_CO_2_Me)­Mo­(CO)_3_Me] in CB8 had a stronger overall effect on the CO release kinetics, increasing *t*_1/2_ from 85 to 420 min, and decreasing the amount released at 6 h from 0.88 to 0.46 mmol of CO per mmol of complex (entry 5 in Table 2).^72^ Based on these comparisons, the inclusion complex **2**@CB7 displays singular behavior since its CO release kinetics profile matches very closely that for the free complex over the whole 3 h course of the Mb assay (Figure 9).

**Table 2:** CO Release Data for 1@CB7 and 2@CB7 Compared with Literature Data for Encapsulated CORMs^a^

entry	compound^b^	medium^c^	[CORM] (μM)^d^	[Mb] (μM)	conditions^e^	CO release^f^ (mol_CO_ mol_M_^–1^)	*t*_1/2_ (min)^g^	ref.
**1**	**1**@CB7	PBS	20	38–47	LED, 400-700 nm, 10 mW cm^–2^	1.04 ± 0.02 (120)	10.0 ± 0.6 (4.3 ± 0.3)	This work
**2**	**2**@CB7	PBS	20	38–47	LED, 400-700 nm, 10 mW cm^–2^	1.26 ± 0.07 (135)	21.1 ± 1.9 (9.9 ± 1.1)	This work
3	[CpMo(CO)_3_Me]@CB7	PBS/DMSO	20	40	UV (365 nm), 2.5 mW cm^–2^	1.0 (30)	10.0 (5.0)	^63^
4	[CpMo(CO)_3_Me]@β-CD	PBS/DMSO	20	40	UV (365 nm), 2.5 mW cm^–2^	0.7 (30)	13.2 (5.5)	^63^
5	[(η^5^-C_5_H_4_CO_2_Me)-Mo(CO)_3_Me]@CB8	PBS/DMSO	20	33	in the dark	0.14 (360)	-	^72^
6		PBS/DMSO	20	36	UV (365 nm), 2.5 mW cm^–2^	0.46 (360)	∼420 (52)	^72^
7	[CpMo(CO)_3_Me]@CB8	PBS/DMSO	20	39	in the dark	0.31 (270)	(165)	^72^
8	P(CD/FpMSt)	H_2_O/DMSO	ns^h^	ns	LED, 405 nm, 30 mW cm^–2^	0.57 (6)^i^	4.0 (2.3)	^84^
9	CORM-1@PLAF	PB	20	ns	UV (365 nm), 1.4 mW cm^–2^	ns	19.7 (10.9)	^85^
10	[Mn(CO)_3_(bpy)(PPh_3_)](ClO_4_)	PBS	2	6	Vis (FL), 600 lm	1.18 (120)^i^	16.5 (8.3)	^86^
11	[Mn(CO)_3_(bpy)(PPh_3_)](ClO_4_)@SFN	PBS	2	6	Vis (FL), 600 lm	1.58 (120)^i^	16.5 (8.3)	^86^
12	ALF795	HEPES	20	∼40	UV (365 nm), 8 mW cm^–2^	1.5 (165)^i^	30 (15.2)	^87^
13	ALF795@LDH	HEPES	20	∼40	UV (365 nm), 8 mW cm^–2^	1.26 (195)	48 (25.6)	^87^
14	Mo(CO)_6_@UiO-66(Hf)	HEPES	10	6–7	UV (365 nm), 15 W	0.42 (270)^i^	(114)	^88^
15	ALF472	PBS	10	10	Vis (FL), 600 lm	0.8 (24 h)^i^	567 (300)	^89^
16	ALF472@bio-MOF-1	PBS	10	10	Vis (FL), 600 lm	0.82 (24 h)^i^	415 (263)	^89^
17	ALF472@SBA-15-SO_3_	PBS	10	10	Vis (FL), 600 lm	0.48 (24 h) ^i^	24 h (12 h)	^89^
18	ALF472@MCM-41-SO_3_	PBS	10	10	Vis (FL), 600 lm	0.65 (24 h) ^i^	934 (456)	^89^
19	ALF472@Al-MCM-41	PBS	10	10	Vis (FL), 600 lm	0.29 (24 h)	(530)	^90^
20	ALF794	HEPES	10	10	in the dark	0.12 (24 h)	-	^91^
21		HEPES	10	10	UV (365 nm)	0.95 (150)	49 (25)	^91^
22	ALF794@[Zn_2_(dhtp)]	HEPES	10	10	UV (365 nm)	0.33 (180)	(79)	^91^
23	ALF794	PBS	10	10	Vis (FL), 600 lm	0.69 (24 h)	400 (175)	^92^
24	ALF794@[Al(OH)(SDC)]*_n_*	PBS	10	10	Vis (FL), 600 lm	0.24 (24 h)	(∼24 h)	^92^

a CO release determined by myoglobin assays at 37 °C. Apart from entry 8, all CORMs were noncovalently conjugated with the carrier.

b β-CD = β-cyclodextrin; P­(CD/FpMSt) = polymethylstyrene derivatized with Cp_2_Fe_2_(CO)_4_@β-CD inclusion complexes; CORM-1 = [Mn_2_(CO)_10_]; PLAF = nanoporous polylactide fibers; bpy = 2,2′-bipyridine; SFN = silk fibroin nanoparticles; ALF794 = [Mo­(CO)_3_(CNCMe_2_CO_2_H)_3_]; ALF795 = [Mo­(CO)_3_(CNCH_2_CO_2_H)_3_]; LDH = layered double hydroxide; UiO-66­(Hf) = Hf-based UiO-66 MOF; ALF472 = [Mn­(1,4,7-triazacyclononane)­(CO)_3_]­Br; bio-MOF-1 = (NH_2_(CH_3_)_2_)_2_[Zn_8_(adeninate)_4_(4,4′-biphenyldicarboxylate)_6_]; dhtp = 2,5-dihydroxyterephthalate; H_2_SDC = 4,4′-stilbenedicarboxylic acid.

c PBS = phosphate buffered saline, PB = phosphate buffer, HEPES = 4-(2-hydroxyethyl)­piperazine-1-ethanesulfonic acid.

d Actual CORM concentration or the equivalent concentration if it was dissolved.

e Vis (FL) = visible light irradiation with fluorescent lamp.

f Time in minutes (or hours, if indicated) in parentheses.

g Values of *t*_1/2_ are defined as the time taken for a CORM with a concentration of X μM to produce an MbCO concentration of X/2 μM. Values of *t*_1/4_ are given in parentheses.

h ns = not specified.

i No MbCO formation for assays performed in the dark.

For the examples listed in Table 2, only two host–guest systems (other than **2**@CB7) yielded *t*_1/2_ values that were equal to or greater than those for the free CORMs. In the first case, a *t*_1/2_ value of 16.5 min was obtained for the visible light (fluorescent lamp)-activated release of CO from both free [Mn­(CO)_3_(bpy)­(PPh_3_)]­(ClO_4_) and a hybrid SFN system (entries 10 and 11).^86^ Within the same time frame (2 h), the SFN hybrid system liberated more CO (1.58 mmol of CO per mmol of encapsulated complex) than the free CORM (1.18 equiv of CO), which was thought to be due to the existence of a charge transfer process from the SFNs to the encapsulated manganese complex. In the second case, encapsulation of [Mn­(1,4,7-triazacyclononane)­(CO)_3_]Br into a zinc-adeninate MOF (bio-MOF-1) led to a decrease in *t*_1/2_ for visible light-activated CO release from 567 to 415 min (entries 15 and 16).^89^ The enhanced CO release rate was attributed to the degradation of the MOF matrix and the concomitant release of adenine and the manganese CORM into solution. Referring to Table 2, it is noteworthy that for CO-releasing materials based on inorganic or metal–organic carriers (entries 13, 14, 16–19, 22 and 24), CO release amounts only exceeded 1 mmol of CO per mmol of encapsulated complex in one case, namely for [Mo­(CO)_3_(CNCH_2_CO_2_H)_3_] intercalated into an LDH (entry 13).^87^ In terms of the amount of CO liberated, this material matched **2**@CB7, with both compounds leading to 1.26 mmol of CO per mmol of encapsulated complex. However, the LDH hybrid system required UV light (365 nm) for photodecarbonylation, and CO release was slower with *t*_1/2_ = 48 min. Another important point to take from the data in Table 2 for encapsulated complexes is that **2**@CB7 has the highest CO releasing efficiency of 63%.

Irradiation of **2**@CB7 in a stepwise manner (15 min irradiation, 10 min dark) showed that CO release could be triggered “on-demand” (Figure 10). Thus, CO release completely stopped when visible light irradiation was suspended and the vial was kept in the dark. Renewed bursts of CO release occurred after reinitiating irradiation. The on/off switchability was sustained over four cycles despite a reduction in the CO release rate and the net liberation of just over one equivalent of the gas. Indeed, the total amount of CO released after four 15 min irradiation steps (1.06 equiv) matched the amount of 1.03 ± 0.09 equiv. released in the normal continuous assay after 60 min (Figure 9). CO delivery systems with this feature, i.e., an efficient temporal control of CO release through an external stimulus, are attractive since the CO concentration can, in principle, be maintained within a therapeutic window (and the risk of CO poisoning is reduced). Although CO release from **2** and **2**@CB7 is activated by visible-light illumination, which has poor tissue penetration, the use of these compounds for the delivery of CO to deep tissues with spatial and temporal control might be achievable through the use of fiber optic technology.^79^ Compound **1**@CB7 also displayed stepwise, phototriggered release of CO over an extended period (Figure 10). Although CO release stopped for the third and fourth darkness intervals, that was not the case for the first and second ones, which is consistent with **1** being a slow CO releaser when incubated in PBS in the dark (Figure 8).

**Figure 10:**
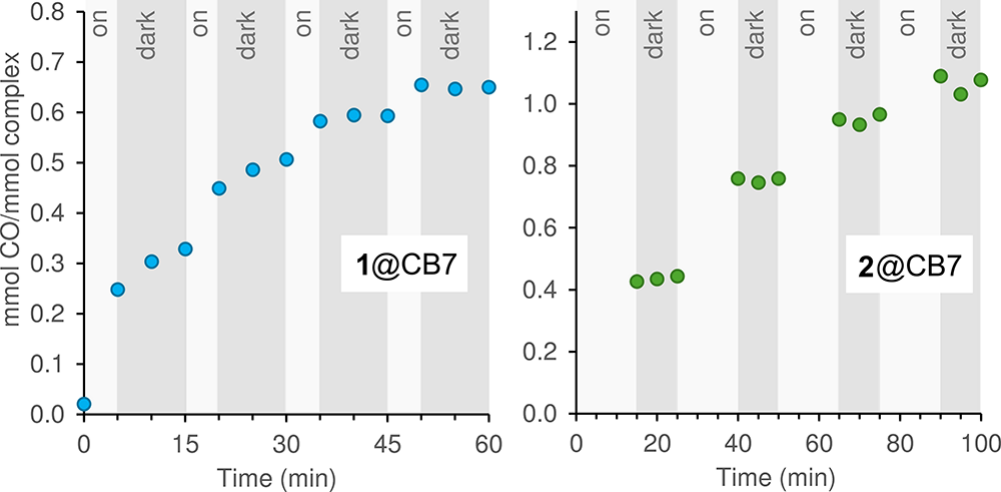
Stepwise CO-release from **1**@CB7 and **2**@CB7 (Mb assay, 37 °C, pH 7.4) using visible light irradiation (λ > 400 nm) for 5 min (**1**@CB7) or 15 min (**2**@CB7) intervals, separated by 10 min darkness intervals.

## Conclusions

The current study has demonstrated that two iron­(II) cyclopentadienyl carbonyl complexes are visible light activatable CORMs (photoCORMs). We investigated the encapsulation of the molecules in CB7 as a first step in the development of formulations for improved photoCORM physical stability, bioavailability and delivery. The isolation of true inclusion compounds in the solid-state was confirmed by ATR-IR, Raman and ^13^C­{^1^H} CP MAS NMR spectroscopies, single-crystal and powder XRD, and TGA. The single-crystal structure of **2**@CB7 is remarkable in revealing two distinct, coexisting host:guest inclusion geometries, one with the Cp ring inside the cavity, and the other with the ring outside the cavity at the level of the carbonyl-lined portal. Binding is stabilized by hydrophobic effects and very weak C–H···O hydrogen bonds. TGA demonstrates that CB7 imparts significant thermal stability to both photoCORMs. CO release studies using the myoglobin assay show that the photoactivatable properties of the dicarbonyl complexes are largely retained in the inclusion compounds.

This work has consequently shown that cucurbiturils are suitable as molecular containers for iron­(II) half-sandwich photoCORMs, with potential applications as anti-inflammatory, antibacterial and anticancer agents, among others. The full encapsulation of the complexes, confirmed crystallographically for **2**@CB7, is likely to protect them to some degree from premature degradation in the body (e.g., through interaction with biological nucleophiles), thus enhancing bioavailability and cellular uptake. Based on the results obtained, the inclusion complex **2**@CB7 may be the best candidate for biological studies, owing to the stability of the photoCORM in aqueous media (in the dark), and the ESI-HRMS and ^1^H NMR studies which verified the formation and stability of a 1:1 inclusion complex in aqueous solution. Future work may first look at the activity of the compounds in *in vitro* models of inflammation and vasorelaxation, for example, which have been classically used in much of the research on CORMs. Regarding the anticancer potential, the *in vitro* antiproliferative activity of the systems could be tested against cancer (e.g., breast, ovarian, colon) and noncancer cell lines. Platinum anticancer drugs such as cisplatin and oxaliplatin, and their known inclusion complexes with CB7, could be used as positive controls. For **2**@CB7, the possibility of controlling the photodecarbonylation in an “on-and-off” manner could be useful in determining whether the photoinduced release of CO can exert some activity complementary to iron-mediated cytotoxicity.

## Experimental Section

### Materials and Methods

CHN Microanalyses were performed using a Leco TruSpec CHNS 630–200–200 instrument. ICP-OES analyses (for Fe) were performed at the Central Analysis Laboratory, University of Aveiro, using a Horiba JobinYvon Activa M spectrometer. PXRD data were collected at rt on a Malvern Panalytical Empyrean diffractometer equipped with a spinning flat sample holder and a PIXcel 1D detector set at 240 nm from the sample, in a Bragg–Brentano para-focusing optics configuration (45 kV, 40 mA). CuK_α1,2_ X-radiation (λ_1_ = 1.540598 Å, λ_2_ = 1.544426 Å) filtered with nickel foil was used. Samples were step-scanned from 3 to 70° (2θ) with 0.026° 2θ steps and a counting time of 50 s per step. TGA studies were performed using a Hitachi STA 300 system at a heating rate of 5 °C min^–1^ under either a nitrogen atmosphere or air. ATR FT-IR spectra were measured on a Bruker Tensor 27 spectrometer equipped with a Specac Golden Gate Mk II ATR accessory having a diamond top plate and KRS-5 focusing lenses (resolution 4 cm^–1^, 256 scans). FT-Raman spectra were recorded on a Bruker MULTIRAM instrument equipped with a Ni:YAG laser with an excitation wavelength of 1064 nm (resolution 4 cm^–1^, 1000 scans). Solid-state ^13^C­{^1^H} CP MAS NMR spectra were recorded on a Bruker Avance III 400 spectrometer (9.4 T) at 100.62 MHz with 3.7 μs ^1^H 90° pulses, 3500 ms contact time, spinning rate of 10–12 kHz, and 5 s recycle delays. Samples were loaded into rotors under argon in a glovebox. ^1^H solution NMR spectra were obtained using Bruker Avance III HD 500 MHz or (for studies of host:guest binding) JEOL 500 MHz spectrometers. Centrifugation was performed using a Hettich Zentrifugen Rotofix 32A centrifuge, working at a speed of 6000 rpm, with samples loaded into 25 mL Falcon tubes. UV–vis spectra were collected on Cintral 303 (for stability studies in solution) and GBC 918 (for the Mb assays) spectrophotometers. For the stability studies and Mb assays, irradiation with visible light was performed using an Aspire LED Floodlight lamp (50 W, 6000 K, 5500 lm, 230 V, 50 Hz, 230 mA, Beam Angle 120°, cold white).

Starting materials and chemicals were purchased from commercial suppliers and used as received. For synthesis: Cyclopentadienyliron dicarbonyl dimer (99%, Thermo Scientific Chemicals), hydrochloric acid (≥37.0%, Fluka), K-selectride (Sigma-Aldrich, 1.0 M in THF), anhydrous absolute ethanol (Carlo Erba), and 2-chloroacetamide (>98%, Alfa Aesar). Anhydrous THF (≥99.9%, Sigma-Aldrich), chloroform (≥99.8%, Sigma-Aldrich) and toluene (≥99.8%, Riedel-de Haën) were stored over 4 Å molecular sieves. For the Mb assays: Equine skeletal muscle (95–100%, lyophilized powder), sodium dithionite, PBS tablet (to prepare 10 mM solution), and carbon monoxide (99.9%) were obtained from Sigma-Aldrich, and Alphagaz Nitrogen type 1 (99.9%) was purchased from AirLiquide. The synthesis of CB7 with the approximate composition C_42_H_42_N_28_O_14_·14H_2_O·0.5­(CH_3_COCH_3_) was described previously.^48^

#### Synthesis of [CpFe­(CO)_2_Cl] (**1**)

This synthesis of **1** was performed according to the literature with minor modifications.^93^ Concentrated HCl (12 M, 5.5 mL) was added, under nitrogen, to a suspension of cyclopentadienyliron dicarbonyl dimer (0.54 g, 1.51 mmol) in ethanol (30 mL), and the mixture was stirred for 4 days at rt. The solvent was removed under reduced pressure, producing a residue that was purified by sublimation at 85 °C, affording **1** as a red solid (0.13 g, 40%). Anal. Calcd for C_7_H_5_ClFeO_2_: C, 39.58; H, 2.37. Found: C, 39.67; H, 2.74. FT-IR (ATR, cm^–1^): 3116 (w), 3083 (w), 2529 (vw), 2485 (vw), 2039 (s), 1980 (vs), 1757 (m), 1423 (m), 1358 (w), 1288 (vw), 1262 (w), 1203 (vw), 1005 (m), 964 (vw), 875 (m), 840 (s), 704 (vw), 650 (w), 595 (s), 564 (s), 527 (s), 478 (s), 438 (m), 417 (w), 375 (w). FT-Raman (cm^–1^): 3128 (m), 3083 (m), 2046 (vs), 1991 (vs), 1428 (m), 1361 (w), 1116 (vs), 1069 (w), 878 (w), 841 (w), 480 (w), 419 (m), 356 (vs), 302 (vs), 165 (w), 152 (m), 115 (vs). ^1^H NMR (CDCl_3_, 500 MHz): δ (ppm) = 5.06 (s, 5H, Cp). ^13^C­{^1^H} NMR (CDCl_3_, 100 MHz): δ (ppm) = 211.7 (Fe-CO), 84.9 (Cp).

#### Synthesis of [CpFe­(CO)_2_CH_2_CONH_2_] (**2**)

The synthesis of **2** was performed according to the literature with minor modifications.^94,95^ Cyclopentadienyliron dicarbonyl dimer (1.01 g, 2.85 mmol) was added to a solution of K-selectride (1.0 M) in THF (6.6 mL) under a nitrogen atmosphere. The mixture was refluxed for 4 h, and the resultant orange precipitate (K^+^[CpFe­(CO)_2_]^−^) was washed with toluene (3 × 20 mL) and dried by blowing nitrogen through the flask. The K^+^[CpFe­(CO)_2_]^−^ was then dissolved in THF (25 mL) and the solution was cooled to –78 °C, at which point a solution of 2-chloroacetamide (0.52 g, 5.60 mmol) in THF (20 mL) was added. The brown mixture was stirred overnight and allowed to warm to rt. After removing the solvent under reduced pressure, the residue was extracted with chloroform (2 × 20 mL), and the solution was evaporated to dryness under reduced pressure. The resultant brown solid was then extracted with diethyl ether (30 mL). Evaporation of the diethyl ether solution to dryness under reduced pressure gave **2** as a dark-yellow solid (0.43 g, 32%). Anal. Calcd for C_9_H_9_FeNO_3_: C, 45.99; H, 3.86; N, 5.96. Found: C, 45.72; H, 4.32; N, 6.06. FT-IR (ATR, cm^–1^): 3473 (w), 3381 (m), 3181 (m), 3114 (w), 2979 (w), 2947 (w), 2005 (s), 1934 (s), 1632 (m), 1587 (s), 1441 (m), 1430 (w), 1354 (m), 1153 (m), 1117 (w), 1097 (w), 1071 (w), 1016 (w), 1007 (w), 915 (w), 883 (w), 841 (w), 826 (m), 774 (w), 755 (w), 625 (m), 603 (w), 581 (w), 560 (s), 506 (w), 480 (w), 449 (m), 387 (w), 361 (w). FT-Raman (cm^–1^): 3122 (w), 3099 (w), 2941 (w), 2016 (m), 2006 (m), 1956 (s), 1946 (s), 1566 (w), 1431 (w), 1358 (w), 1155 (w), 1118 (s), 1095 (w), 1071 (m), 1051 (m), 916 (w), 602 (w), 507 (w), 447 (w), 425 (w), 378 (m), 357 (vs), 205 (w), 188 (w), 148 (w), 111 (s). ^1^H NMR (CDCl_3_, 500 MHz): δ (ppm) = 4.92 (s, Cp), 1.58 (s, CH_2_). ^13^C­{^1^H} NMR (CDCl_3_, 100 MHz): δ (ppm) = 216.3 (Fe-CO), 186.2 (CONH_2_), 86.1 (Cp), –1.5 (CH_2_).

### General Procedure for the Synthesis of the Inclusion Compounds

A solution of **1** or **2** (0.28 mmol) in ethanol (2 mL) was added to CB7 (0.40 g, 0.28 mmol). Finally, Milli-Q water (20 mL) was added, and the resultant suspension was shielded from light and stirred for 24 h at rt. The mixture was then centrifuged for 15 min with a speed of 6000 rpm. After decanting the mother liquor, the solid was vacuum-dried for 2 h at rt.

#### **1**@CB7

Light-orange solid (0.20 g, 43%). Anal. Calcd for C_42_H_42_N_28_O_14_·C_7_H_5_ClFeO_2_·15H_2_O: C, 35.76; H, 4.72; N, 23.83; Fe, 3.39. Found: C, 35.70; H, 3.80; N, 23.24; Fe, 3.66. FT-IR (ATR, cm^–1^): 3438 (m, bd), 3006 (vw), 2934 (vw), 2051 (m), 2005 (m), 1715 (s), 1463 (s), 1418 (m), 1375 (m), 1318 (m), 1295 (vw), 1227 (s), 1185 (s), 1154 (vw), 1029 (vw), 965 (s), 823 (w), 797 (s), 754 (m), 668 (m), 626 (vw), 605 (vw), 567 (vw), 544 (w), 441 (w), 362 (m). FT-Raman (cm^–1^): 3112 (vw), 2951 (s), 2055 (m), 2009 (m), 1738 (m), 1424 (s), 1385 (s), 1327 (m), 1282 (w), 1233 (w), 1205 (w), 1118 (m), 1045 (w), 973 (w), 902 (m), 829 (vs), 754 (m), 657 (m), 440 (vs), 360 (m), 324 (m), 289 (w), 191 (w). ^13^C­{^1^H} CP MAS NMR (100.62 MHz): δ (ppm) = 212.2 (Fe-CO), 156.1 (C=O), 88.6 (C_5_H_5_), 71.0 (CH), 52.8 (CH_2_).

#### **2**@CB7

Light-yellow solid (0.24 g, 53%). Anal. Calcd for C_42_H_42_N_28_O_14_·C_9_H_9_FeNO_3_·15H_2_O: C, 36.72; H, 4.89; N, 24.35; Fe, 3.35. Found: C, 36.57; H, 4.04; N, 24.13; Fe, 2.96. FT-IR (ATR, cm^–1^): 3393 (m, bd), 3007 (vw), 2932 (vw), 2019 (m), 1964 (m), 1716 (s), 1462 (s), 1419 (m), 1372 (m), 1316 (m), 1295 (w), 1226 (s), 1183 (s), 1152 (w), 1028 (w), 964 (s), 823 (w), 797 (s), 754 (m), 666 (w), 627 (w), 583 (vw), 439 (w), 358 (m). FT-Raman (cm^–1^): 3124 (w), 2993 (sh), 2944 (s), 2021 (m), 1966 (m), 1744 (m), 1423 (m), 1382 (m), 1325 (w), 1284 (w), 1229 (w), 1202 (w), 1119 (m), 1064 (w), 1044 (m), 974 (w), 901 (m), 830 (s), 754 (m), 710 (vw), 656 (m), 516 (vw), 440 (vs), 367 (s), 289 (w), 192 (m), 95 (m). ^13^C­{^1^H} CP MAS NMR (100.62 MHz): δ (ppm) = 215.6 (Fe-CO), 189.3 (CONH_2_), 156.3 (C=O), 86.3 (Cp), 71.3 (CH), 52.8 (CH_2_), –1.83 (CH_2_).

### Single-Crystal X-ray Diffraction Studies

Single crystals of **2**@CB7 were manually harvested from the crystallization vial and immersed in highly viscous FOMBLIN Y perfluoropolyether vacuum oil (LVAC 140/13, Sigma-Aldrich) to avoid degradation caused by the evaporation of the solvent.^96^ The crystals were mounted on either Hampton Research CryoLoops or MiTeGen MicroLoops, typically with the help of a Stemi 2000 stereomicroscope equipped with Carl Zeiss lenses.

Crystal data was collected at 150(2) K on a RIGAKU XtaLAB Synergy-i equipped with a Mo Kα (λ = 0.71073 Å) PhotonJet-i microsource, a HyPix3000 detector controlled by the CrysAlisPro^97^ software and equipped with an Oxford Cryosystems Series 800 cooler. Diffraction images were processed using the CrysAlisPro software and the data were corrected for absorption by the multiscan absorption correction using spherical harmonics implemented in SCALE3 ABSPACK scaling algorithm.

The structure was solved using the algorithm implemented in SHELXT-2014/5,^98^ which allowed the immediate location of almost all the heaviest atoms composing the asymmetric unit. The remaining missing and misplaced non-hydrogen atoms were located from difference Fourier maps calculated from successive full-matrix least-squares refinement cycles on *F* ^2^ using the latest SHELXL from the 2018/3 release.^99^ All structural refinements were performed using the graphical interface ShelXle.^100^

Hydrogen atoms bound to carbon were placed at their idealized positions using HFIX instructions in SHELXL: 13 for the –CH groups, 23 for the –CH_2_ groups, 33 for the –CH_3_ groups, 43 for cyclopentadienyl (Cp) ring carbon atoms, and 93 for the –NH_2_ groups. These hydrogen atoms were included in subsequent refinement cycles with isotropic thermal displacement parameters (*U*_iso_) fixed at 1.5 × *U*_eq_ (for the –CH_3_ groups), and 1.2 × *U*_eq_ (for the remaining groups) of the parent atoms. Of the 40.65 water molecules of crystallization only 8 were refined with full occupancy (OW3, OW4, OW13, OW30, OW39, OW42, OW47 and OW48). The remaining 32.65 water molecules were highly disordered and refined over 58 different crystallographic positions. One ethanol molecule was disordered and refined with an occupancy rate of 70%.

The last difference Fourier map synthesis showed the highest peak (2.37 eÅ^–3^) and the deepest hole (−2.29 eÅ^–3^) located at 1.15 and 0.10 Å from Fe1 and O34, respectively. All structural drawings were created using Crystal Impact Diamond.^101^ Crystal data and structure refinement details for **2**@CB7 are given in Table S1 in the Supporting Information.

### HRMSn

The formation of **1**@CB7 and **2**@CB7 complexes in aqueous solution was studied using an Orbitrap Elite (Thermo Scientific, Bremen, Germany) mass spectrometer. The mass spectrometer comprises a linear ion trap analyzer with MSn, n = 2–10, and a high field high resolution orbitrap. The system was operated using an ESI ion source (HESI-II). Data were acquired under positive polarity. The flow was 5 μL min^–1^. Ethanolic and aqueous 50 μM or 100 μM solutions of guests were prepared and infused first. Free **1** and **2** were studied using the following parameters: heater temperature 50 °C; sheath gas flow 10 arbitrary units; capillary temperature 70 °C; spray voltage 3.5 kV; S-Lenses RF level, 68%. Scan range was 50–600 *m*/*z*. Complexes were prepared in aqueous solutions in 1:1 stoichiometry using 50 μM or 100 μM concentrations of host and guest. The detection of the CB7 complexes was performed using the following spray and ion optics parameters: heater temperature 40 °C; sheath gas flow 10 arbitrary units; capillary temperature 325 °C; spray voltage 3.5 kV; S-Lenses RF level, 68%. Scan range was 100–1500 *m*/*z*. Fragmentation was performed by CID at the linear trap or by HCD at the collision cell. The data were analyzed using Xcalibur 4.1 (Thermo Fisher Scientific).

### Stability Studies

To assess stability in the solid state, samples of **1**, **2**, **1**@CB7 and **2**@CB7 were placed on a watch glass and exposed to air and ambient light for 28 days. ATR FT-IR spectra were recorded periodically.

The aqueous formation of host-guest complexes and their stability was studied using a 500 MHz JEOL NMR. Additionally, UV–vis spectroscopy was used to assess the stability of complexes **1**, **2**, **1**@CB7 and **2**@CB7 in solution. Stock solutions of each compound (4 mM) were prepared in degassed 10 mM PBS (pH 7.4). Aliquots of each solution were added to a sealed quartz cell (3500 μL) under a nitrogen atmosphere, and 10 mM PBS was added to give a final concentration of 100 μM. The cells were kept either in the dark or under constant visible light irradiation (*E* = 10 mW cm^–2^), at 37 °C, with magnetic stirring for 120 or 180 min. The incubations were interrupted periodically to record absorption spectra in the range of 190–900 nm (scan speed 200 nm min^–1^, slit width 2 nm).

### Mb Assay for the Detection of CO Release

CO release from **1**, **2**, **1**@CB7 and **2**@CB7 was explored using the Mb assay at 37 °C in which the binding of released CO to reduced deoxy-Mb was followed spectrophotometrically through the decrease of the 557 nm band and the concomitant increase of the 540 and 577 nm bands.^71^

Stock solutions of a horse skeletal Mb (∼100 μM) and sodium dithionite (40 mg mL^–1^) were freshly prepared in degassed 10 mM PBS (pH 7.4). The solutions were added in the following order to a sealed quartz cell (3500 μL) under a nitrogen atmosphere: 10 mM PBS (1185 μL), 100 μM Mb (1500 μL), 40 mg mL^–1^ sodium dithionite (300 μL). A spectrum of the resultant solution was recorded to capture the deoxy-Mb profile (0% MbCO). After that, a stock solution of complex **1**, **2**, **1**@CB7 or **2**@CB7 was prepared in degassed 10 mM PBS (pH 7.4). An aliquot (15 μL) of this solution was added to the sealed cell to give a final concentration of 20 μM (based on %Fe (ICP) for the inclusion compounds). The quartz cell was kept either in the dark or under visible light irradiation (λ = 400–700 nm, *E* = 10 mW cm^–2^), at 37 °C, and with constant magnetic stirring at 120 rpm. The assays were conducted for 120 or 180 min, and the incubations were interrupted in intervals of 15 min to measure absorption spectra between 450 and 650 nm, with a scan speed of 200 nm min^–1^ and a slit width of 2 nm. At the end of each assay, CO gas was bubbled through the liquid phase to achieve 100% conversion to MbCO, and a final spectrum was recorded to capture the MbCO profile (100% MbCO). The actual concentration of Mb in each solution (always within the range of 38–47 μM) was determined by using the MbCO extinction coefficient (ε = 15.4 mM^–1^ cm^–1^).^102^ The assays were carried out in triplicate. The spectroscopic data were treated in the standard way by applying a correction at the 510 nm isosbestic point.^71^ For the determination of quarter-lives (*t*_1/4_) and half-lives (*t*_1/2_), the software GraphPad Prism (version 8 for Windows, GraphPad Software, Boston, Massachusetts USA, www.graphpad.com) was used.

## Supplementary Material



## Notes

ATR FT-IR spectra for stability studies in the solid-state, UV–vis spectra for stability studies in the solution, ESI-HRMS and ^1^H NMR spectra, UV–vis spectra for Mb assays, crystal data and structure refinement details for **2**@CB7 (PDF)
