# Supplementary material for: Host–Guest Complexes of Cyclopentadienyl Iron Dicarbonyl (CpFe(CO)2) CO-Releasing Molecules with Cucurbit[7]uril
Source: Organometallics. 2025 Mar 26;44(7):824–39. doi: 10.1021/acs.organomet.4c00469 (PMC12820945; doi:10.1021/acs.organomet.4c00469)
Supplement: Supplementary file 1 [file om4c00469_si_001.pdf]

Supporting Information

Host-Guest Complexes of Cyclopentadienyl Iron Dicarbonyl ($CpFe(CO)_2$) CO-Releasing Molecules with Cucurbit[7]uril

Rodrigo P. Monteiro,[†] Isabel B. Calhau,[†] Ana C. Gomes,[†] Ricardo F. Mendes,[†] Filipe A. Almeida Paz,[†] André D. Lopes,[‡] José P. Da Silva,[‡] Carlos C. Romão,[§] Isabel S. Gonçalves,[†] and Martyn Pillinger[†,*]

† CICECO - Aveiro Institute of Materials, Department of Chemistry, University of Aveiro, Campus Universitário de Santiago, 3810-193 Aveiro, Portugal

‡ Centre of Marine Sciences (CCMAR/CIMAR LA), and Department of Chemistry and Pharmacy, FCT, University of the Algarve, 8005-039 Faro, Portugal

§ Instituto de Tecnologia Química e Biológica António Xavier, Universidade Nova de Lisboa, Avenida da República (EAN), 2780-157 Oeiras, Portugal

*Corresponding author.
E-mail: mpillinger@ua.pt

**Contents**	**page**
**Fig. S1** ATR FT-IR spectra in the solid-state of **1** and **2** after exposure to ambient air and light for different times.	S3
**Fig. S2** ATR FT-IR spectra in the solid-state of **1**@CB7 and **2**@CB7 after exposure to ambient air and light for different times.	S3
**Fig. S3** ESI-HRMS full scan spectrum (positive ESI) of **1** in aqueous solution (100 μM).	S4
**Fig. S4** ESI-HRMS full scan spectrum (positive ESI) of **1** in ethanol (100 μM).	S4
**Fig. S5** ESI-HRMS^2(235) spectrum (positive ESI, CID) of **1** in ethanol (100 μM).	S5
**Fig. S6** ESI-HRMS full scan spectrum (positive ESI) of **2** in water (100 μM).	S5
**Fig. S7** ESI-HRMS^2(236) spectrum (positive ESI, CID) of **2** in water (100 μM).	S6

**Fig. S8** ESI-HRMS full scan spectrum (positive ESI) of **1**@CB7 in water (1:1 stoichiometry, 100 μM).	S6
**Fig. S9** ESI-HRMS^2(699) spectrum (positive ESI, HCD) of **2**@CB7 in aqueous solution (1:1 stoichiometry, 100 μM).	S7
**Fig. S10** ^{1}H NMR spectra (500 MHz) of **2** (1 mM, D_2O) and **2**@CB7 (1:1, 1 mM, D_2O).	S8
**Fig. S11** ^{1}H NMR partial spectra (500 MHz, D_2O) of free **2** (2 mM), and of **2** (2 mM) plus CB7 ([CB7] = 0.5, 1.0 and 2.0 mM).	S8
**Fig. S12** UV-vis spectra of **1** (100 μM) in degassed 10 mM PBS (pH 7.4) obtained over a period of 2 hours, in the dark.	S9
**Fig. S13** UV-vis spectral changes observed upon irradiation of **1** (100 μM) in degassed 10 mM PBS (pH 7.4) with visible light (E = 10 mW cm^{-2}).	S9
**Fig. S14** UV-vis spectral changes observed upon irradiation of **2** (100 μM) in degassed 10 mM PBS (pH 7.4) with visible light (E = 10 mW cm^{-2}).	S10
**Fig. S15** UV-vis spectra of freshly prepared (in the dark) solutions of **1** and **2** (100 μM) in degassed 10 mM PBS (pH 7.4).	S10
**Fig. S16** UV-vis spectral changes observed upon irradiation of **1**@CB7 (100 μM) in degassed 10 mM PBS (pH 7.4) with visible light (E = 10 mW cm^{-2}).	S11
**Fig. S17** UV-vis spectral changes observed upon irradiation of **2**@CB7 (100 μM) in degassed 10 mM PBS (pH 7.4) with visible light (E = 10 mW cm^{-2}).	S11
**Fig. S18** UV-vis spectra of **1** and **1**@CB7 before (0 min) and after (120 min) irradiation of 100 μM solutions in degassed 10 mM PBS (pH 7.4) with visible light (E = 10 mW cm^{-2}).	S12
**Fig. S19** UV-vis spectra of **2** and **2**@CB7 before (0 min) and after (180 min) irradiation of 100 μM solutions in degassed 10 mM PBS (pH 7.4) with visible light (E = 10 mW cm^{-2}).	S12
**Fig. S20** UV-vis spectra for individual Mb assays performed with **1**, **2**, **1**@CB7 and **2**@CB7 either in the dark or under visible light irradiation (λ = 400-700 nm, E = 10 mW cm^{-2}, 37 °C, 0.01 M PBS, pH 7.4).	S13
**Table S1.** Crystal data and structure refinement of **2**@CB7	S14

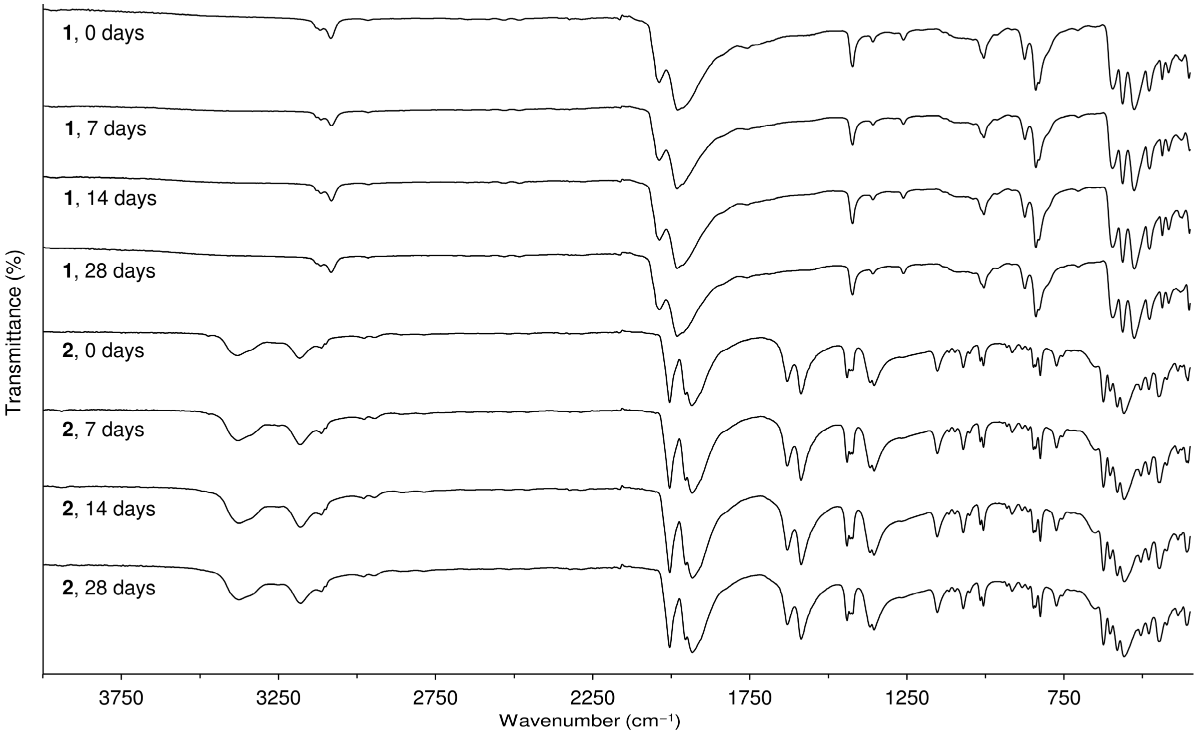

Fig. S1 ATR FT-IR spectra in the solid-state of **1** and **2** after exposure to ambient air and light for different times.

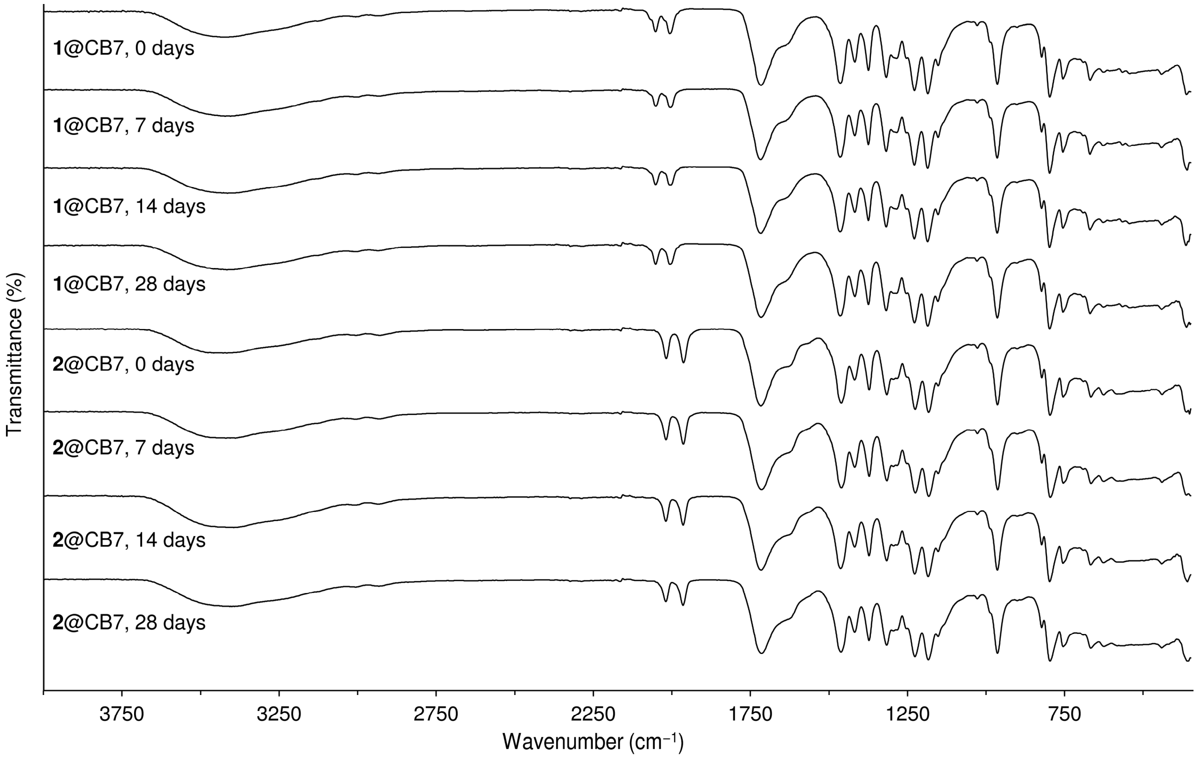

Fig. S2 ATR FT-IR spectra in the solid-state of **1**@CB7 and **2**@CB7 after exposure to ambient air and light for different times.

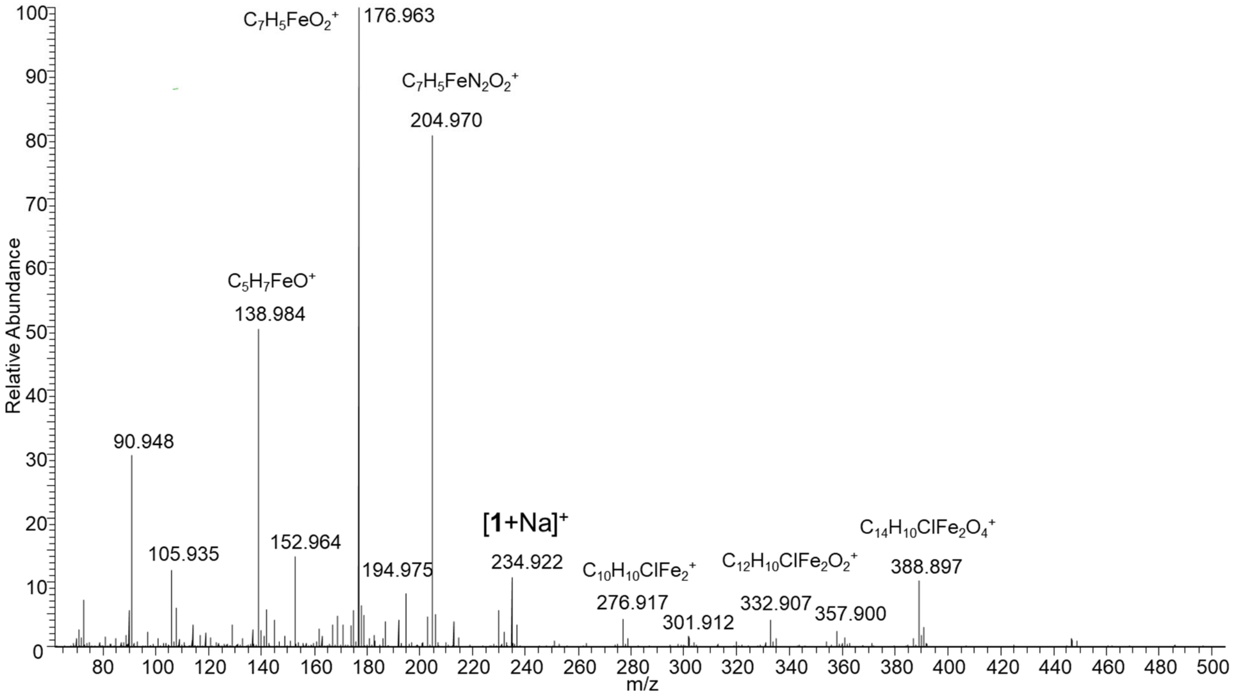

Fig. S3 ESI-HRMS full scan spectrum (positive ESI) of **1** in aqueous solution (100 μM).

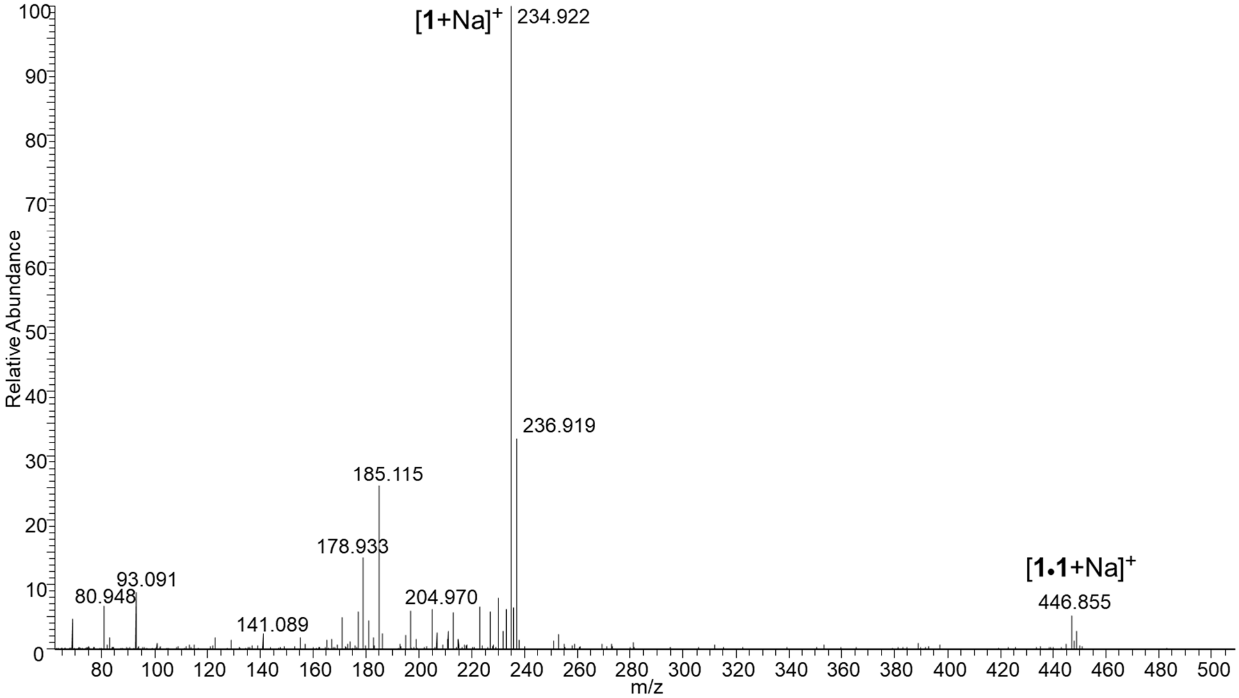

Fig. S4 ESI-HRMS full scan spectrum (positive ESI) of **1** in ethanol (100 μM).

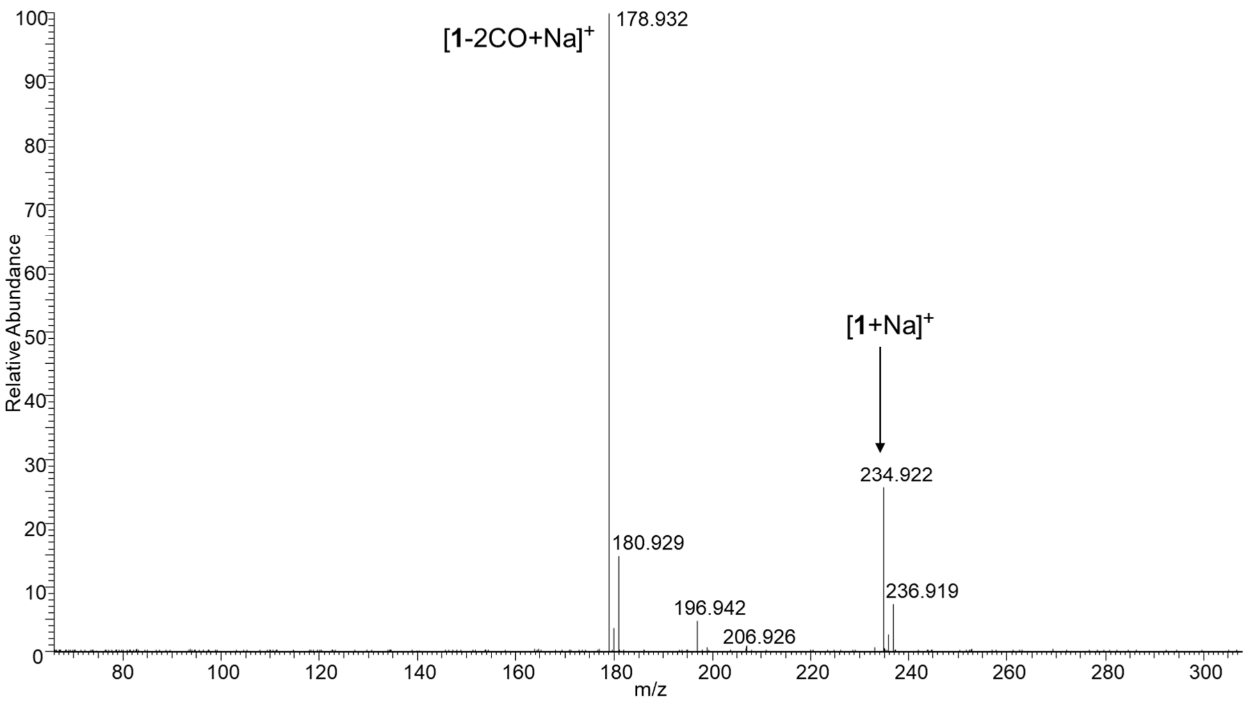

Fig. S5 ESI-HRMS^2(235) spectrum (positive ESI, CID) of **1** in ethanol (100 μM).

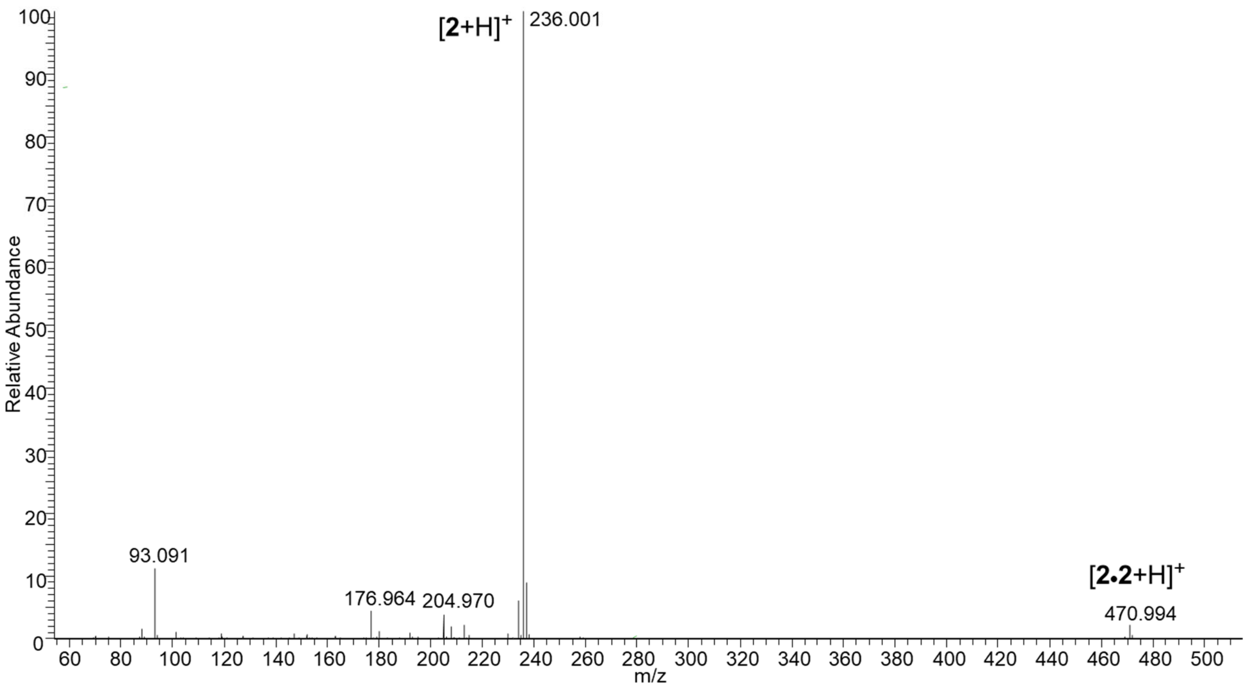

Fig. S6 ESI-HRMS full scan spectrum (positive ESI) of **2** in water (100 μM).

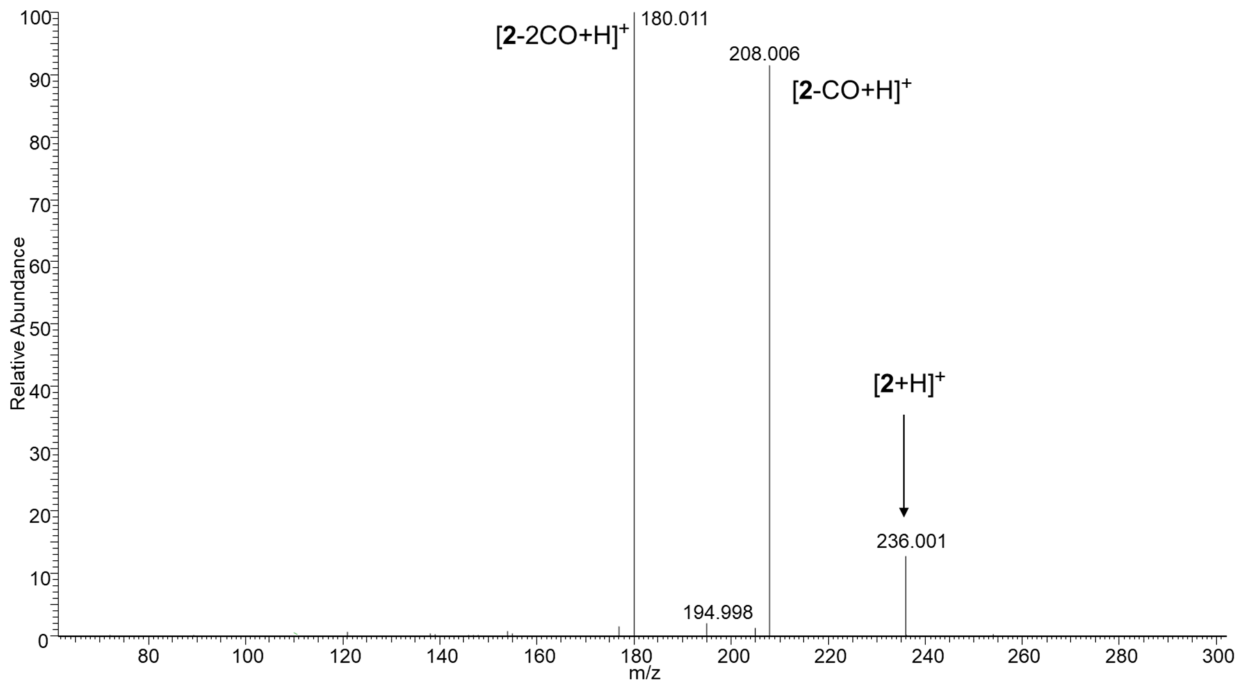

Fig. S7 ESI-HRMS^2(236) spectrum (positive ESI, CID) of **2** in water (100 μM).

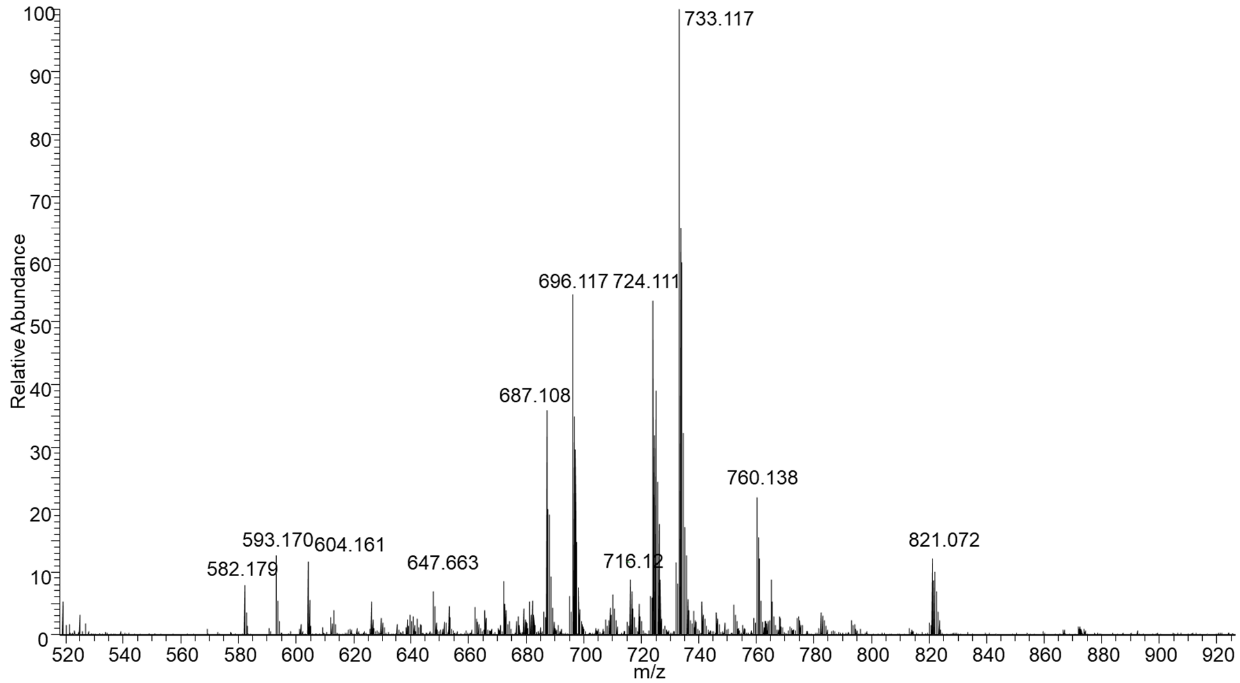

Fig. S8 ESI-HRMS full scan spectrum (positive ESI) of **1**@CB7 in water (1:1 stoichiometry, 100 μM).

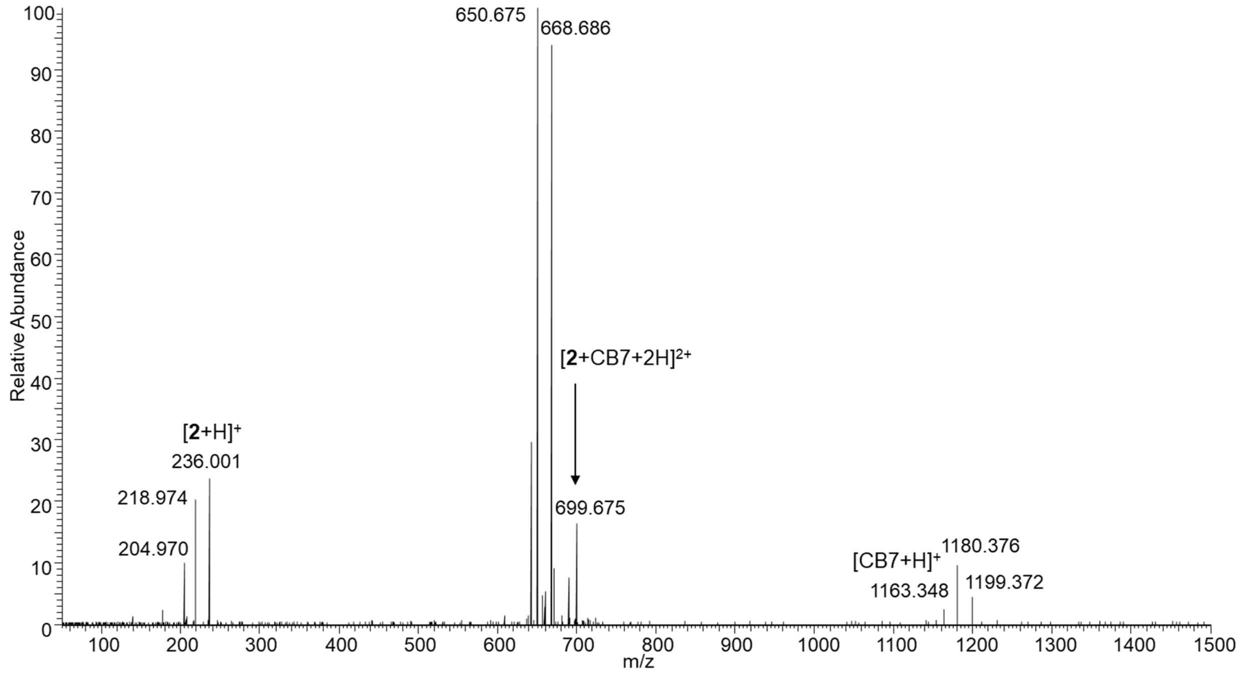

Fig. S9 ESI-HRMS^2(699) spectrum (positive ESI, HCD) of **2**@CB7 in aqueous solution (1:1 stoichiometry, 100 μM).

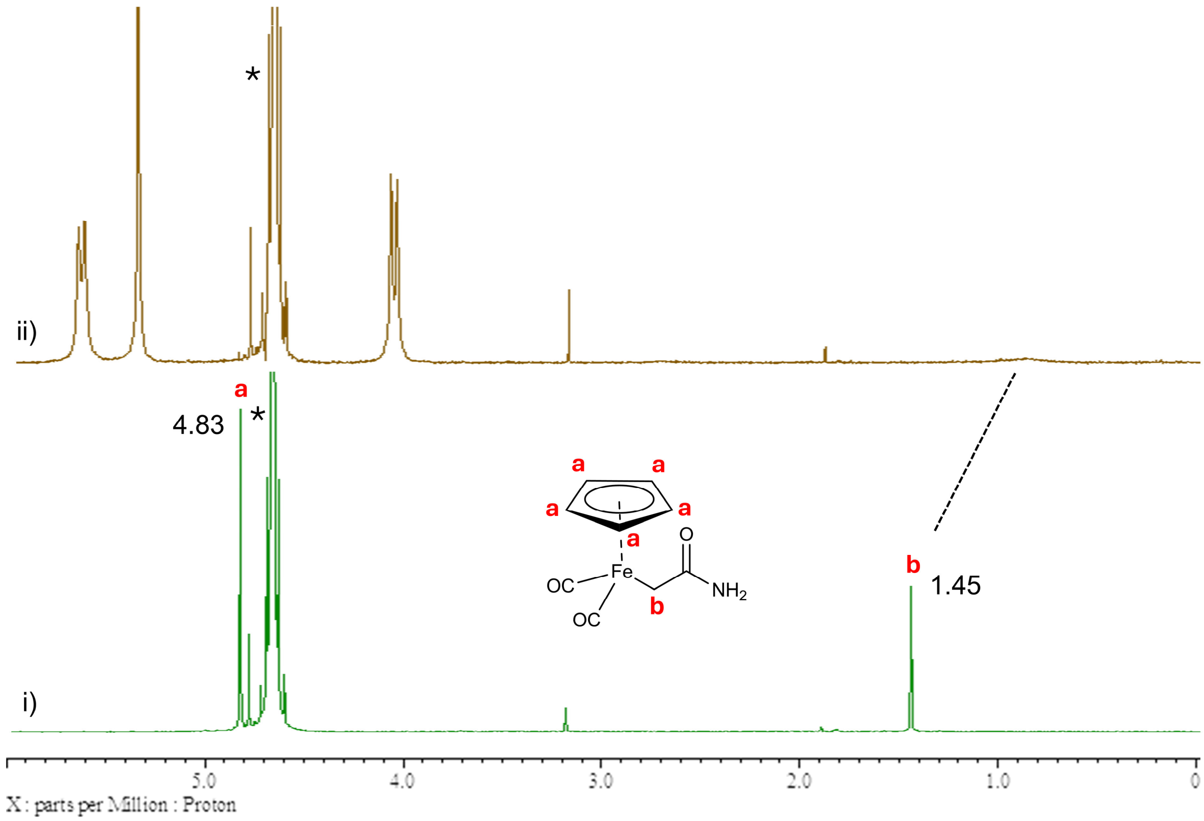

Fig. S10 ^{1}H NMR spectra (500 MHz) of **2** (1 mM, D_2O), i), and **2**@CB7 (1:1, 1 mM, D_2O), ii). The asterisk (*) marks the HOD signals.

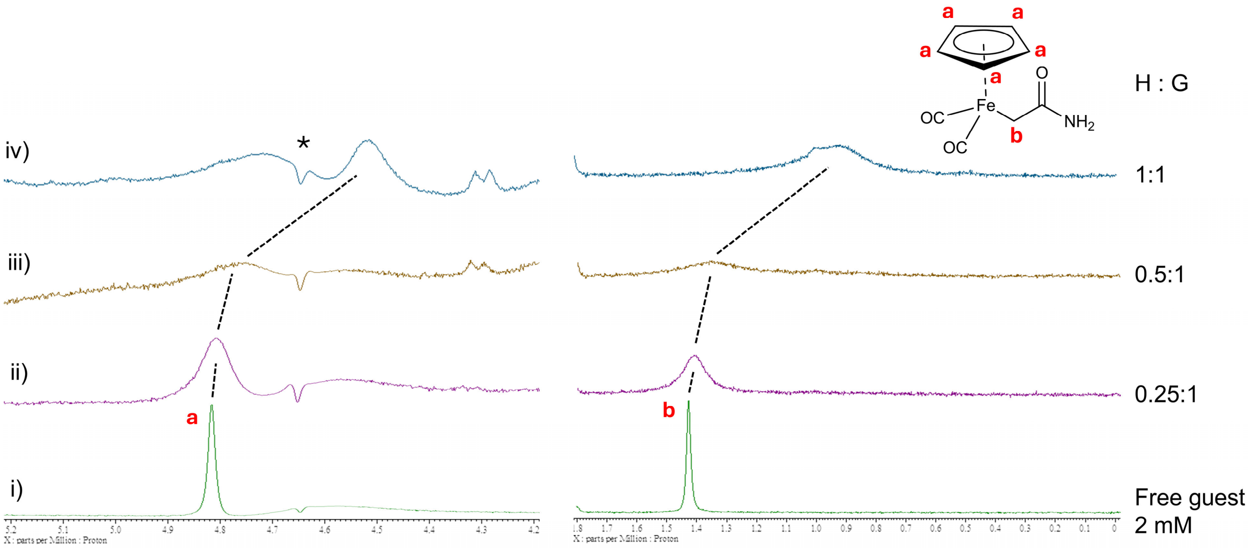

Fig. S11 ^{1}H NMR partial spectra (500 MHz, D_2O) of i) free **2** (2 mM), and of (ii)-iv)) **2** (2 mM) plus CB7 ([CB7] = 0.5, 1.0 and 2.0 mM). Signals of **2** in the presence of CB7 were zoomed. A pulse sequence for water suppression was used. The asterisk (*) marks the residual HOD signal.

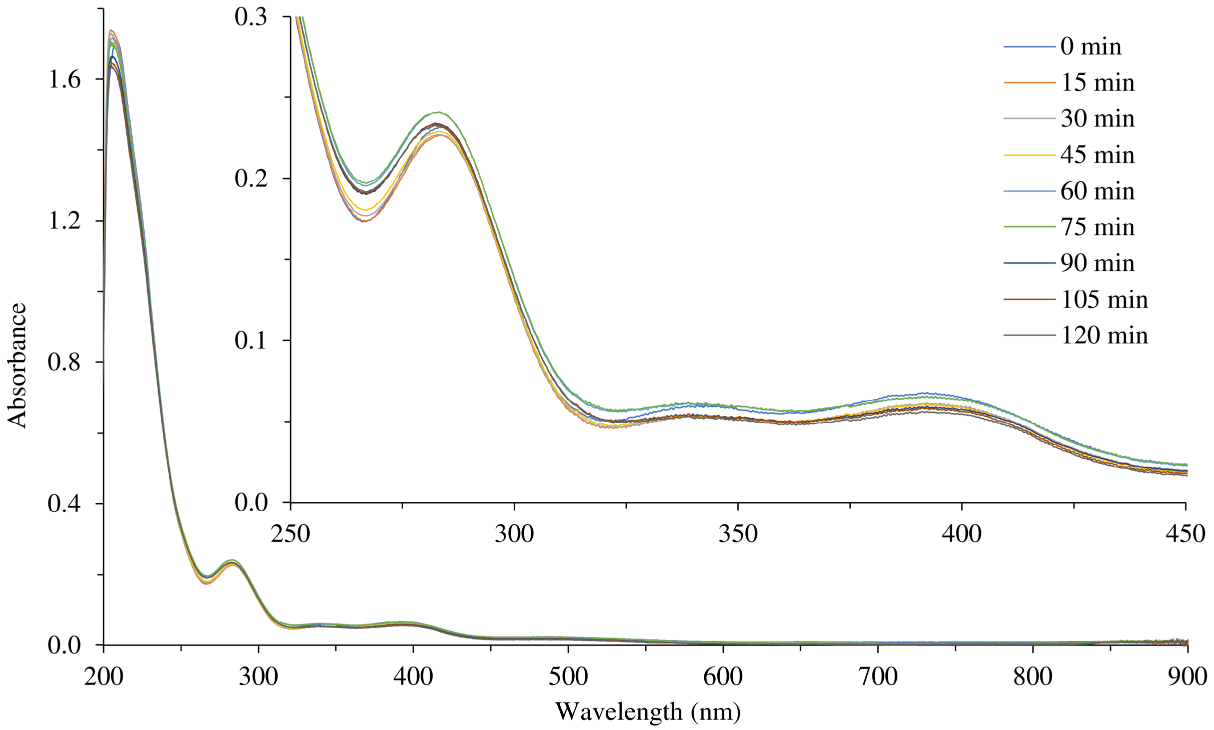

Fig. S12 UV-vis spectra of **1** (100 µM) in degassed 10 mM PBS (pH 7.4) obtained over a period of 2 hours, in the dark.

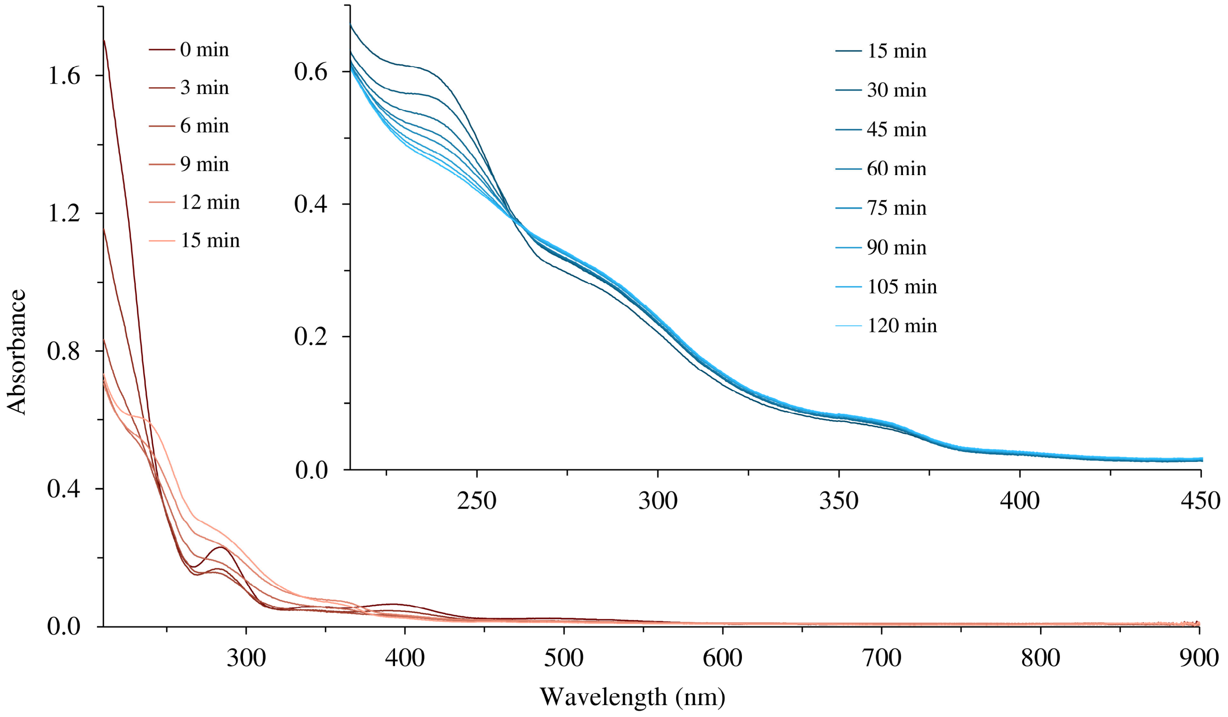

Fig. S13 UV-vis spectral changes observed upon irradiation of **1** (100 µM) in degassed 10 mM PBS (pH 7.4) with visible light (E = 10 mW cm^{-2}).

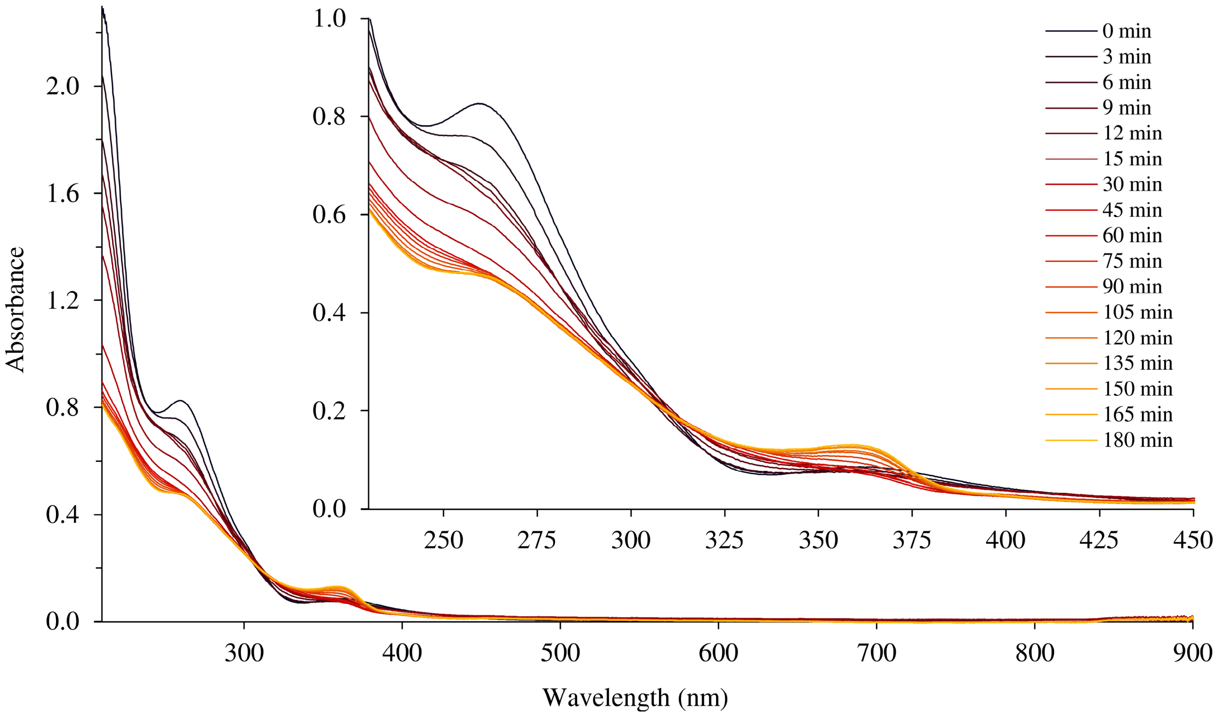

Fig. S14 UV-vis spectral changes observed upon irradiation of **2** (100 µM) in degassed 10 mM PBS (pH 7.4) with visible light (E = 10 mW cm^{-2}).

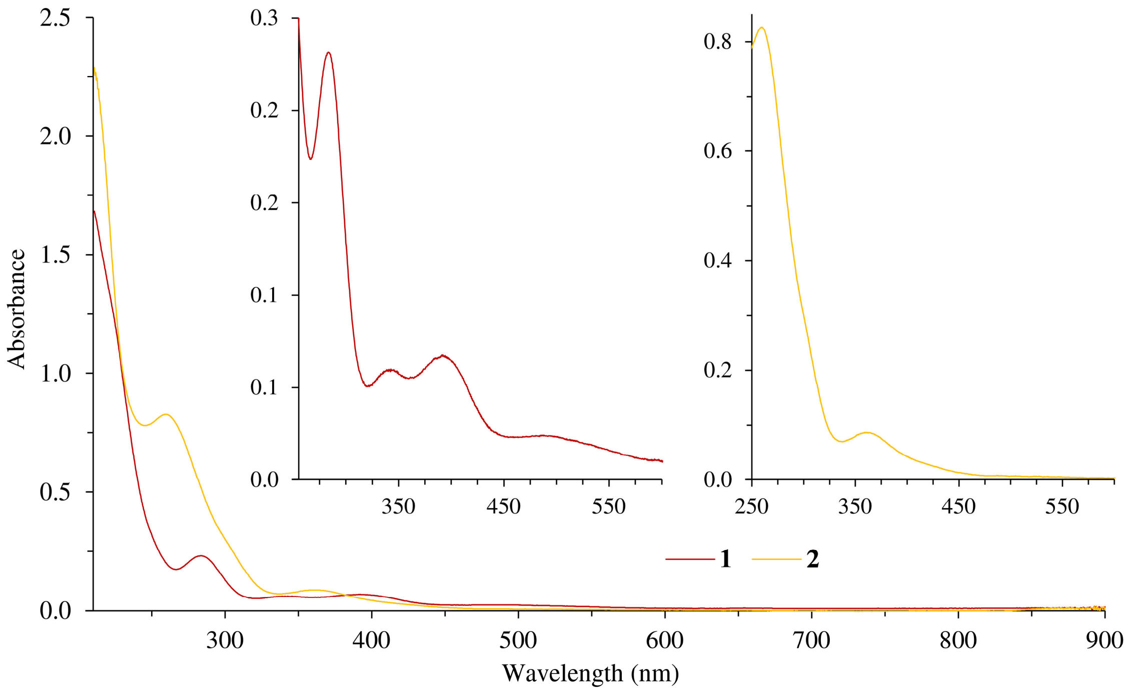

Fig. S15 UV-vis spectra of freshly prepared (in the dark) solutions of **1** and **2** (100 µM) in degassed 10 mM PBS (pH 7.4).

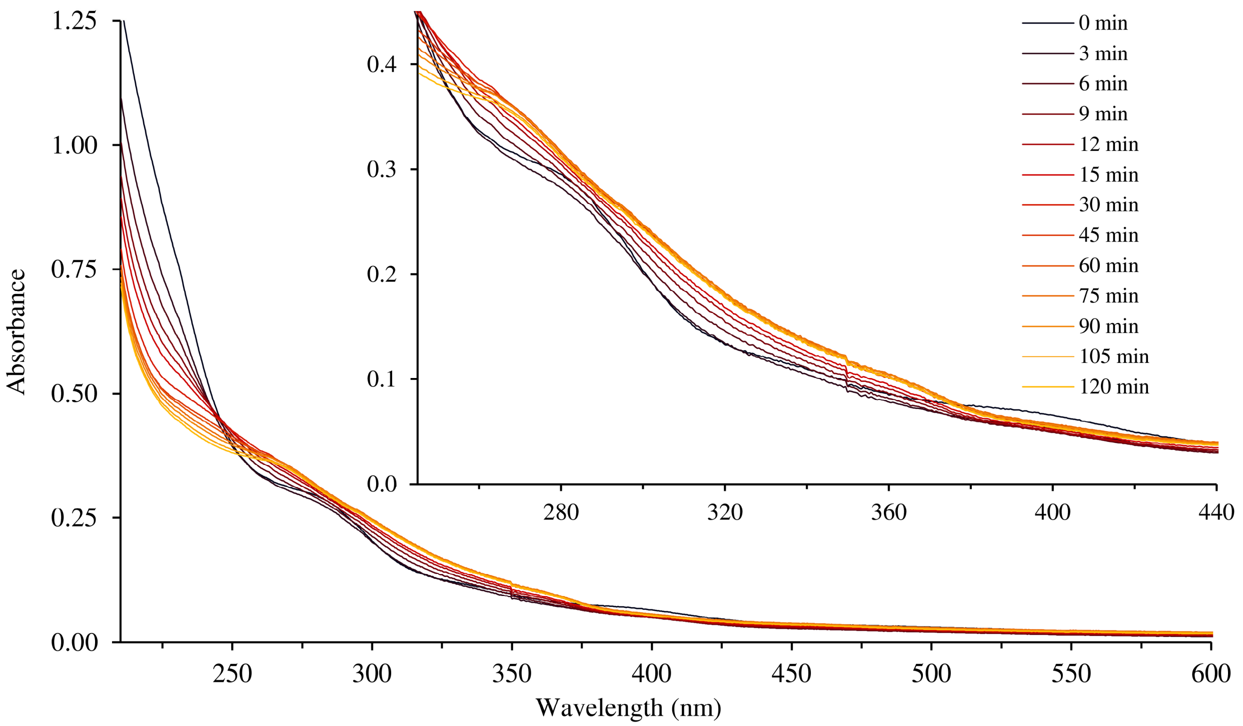

Fig. S16 UV-vis spectral changes observed upon irradiation of **1**@CB7 (100 μM) in degassed 10 mM PBS (pH 7.4) with visible light (E = 10 mW cm^{-2}).

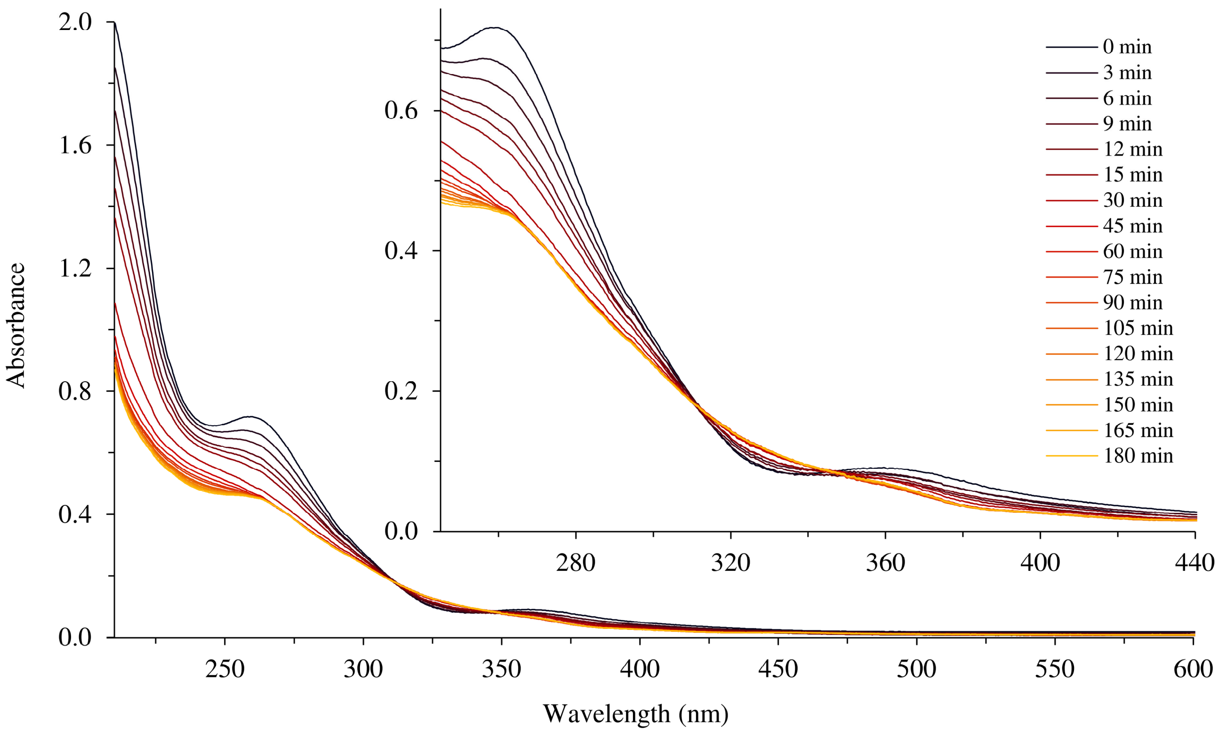

Fig. S17 UV-vis spectral changes observed upon irradiation of **2**@CB7 (100 μM) in degassed 10 mM PBS (pH 7.4) with visible light (E = 10 mW cm^{-2}).

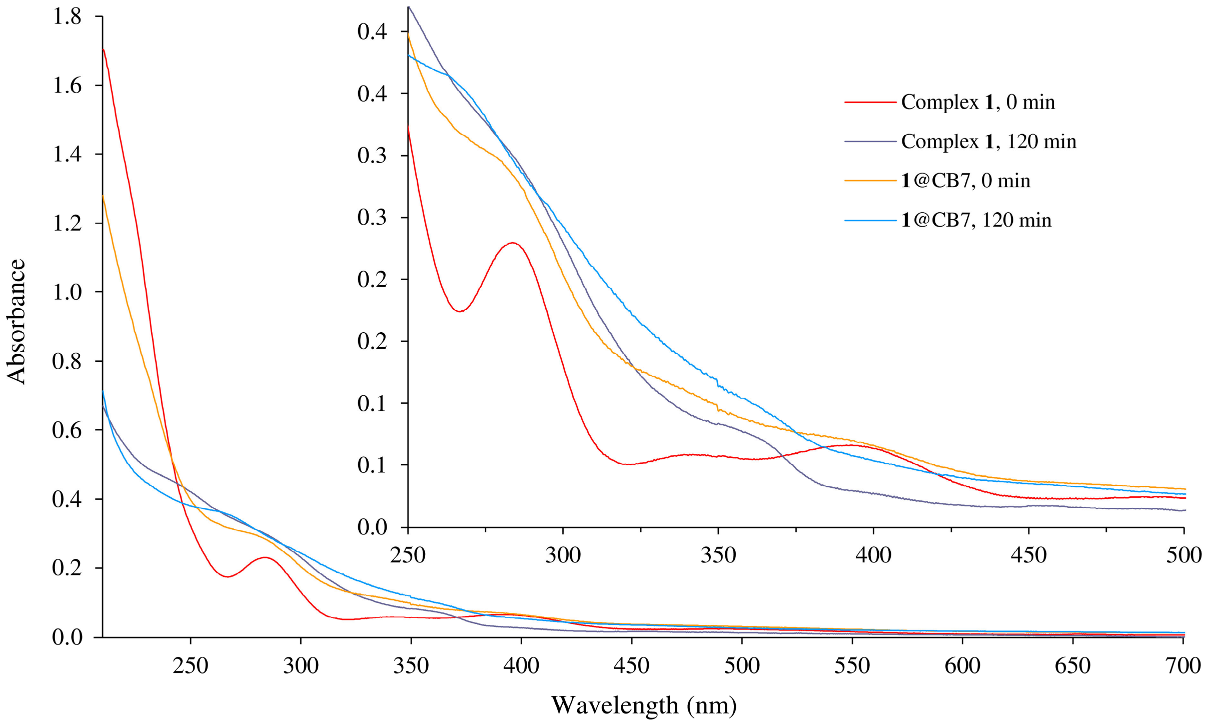

Fig. S18 UV-vis spectra of **1** and **1**@CB7 before (0 min) and after (120 min) irradiation of 100 μM solutions in degassed 10 mM PBS (pH 7.4) with visible light (E = 10 mW cm^{-2}).

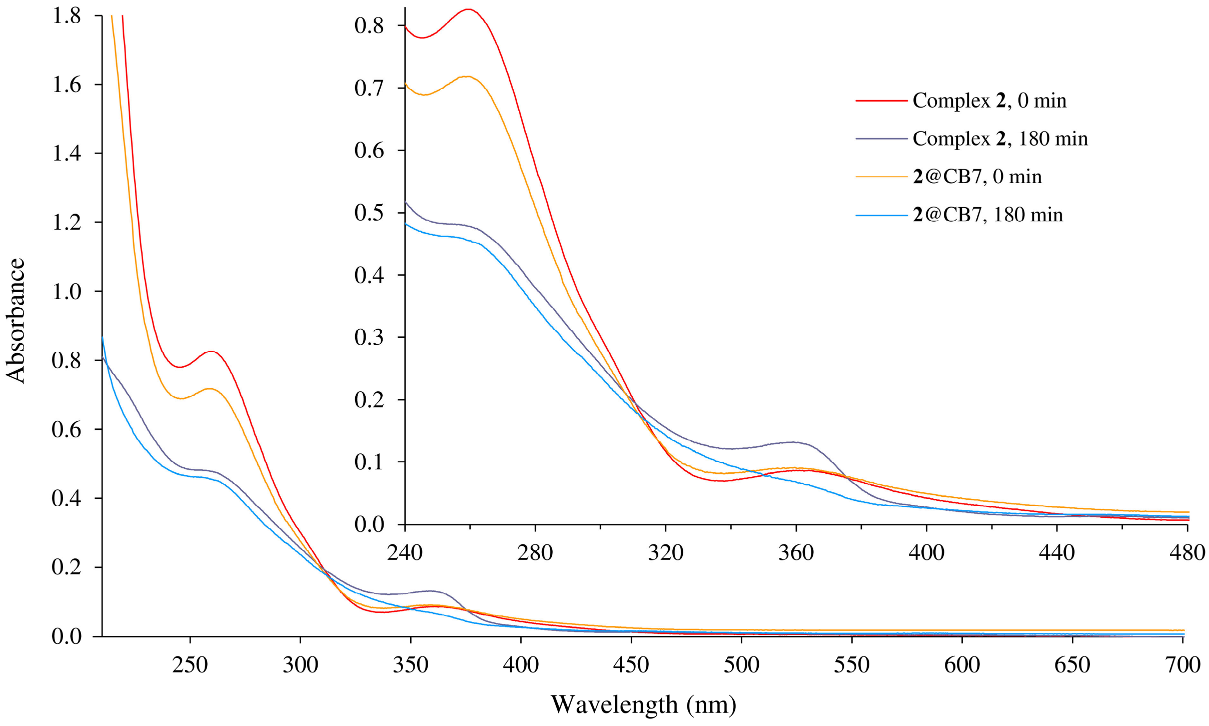

Fig. S19 UV-vis spectra of **2** and **2**@CB7 before (0 min) and after (180 min) irradiation of 100 μM solutions in degassed 10 mM PBS (pH 7.4) with visible light (E = 10 mW cm^{-2}).

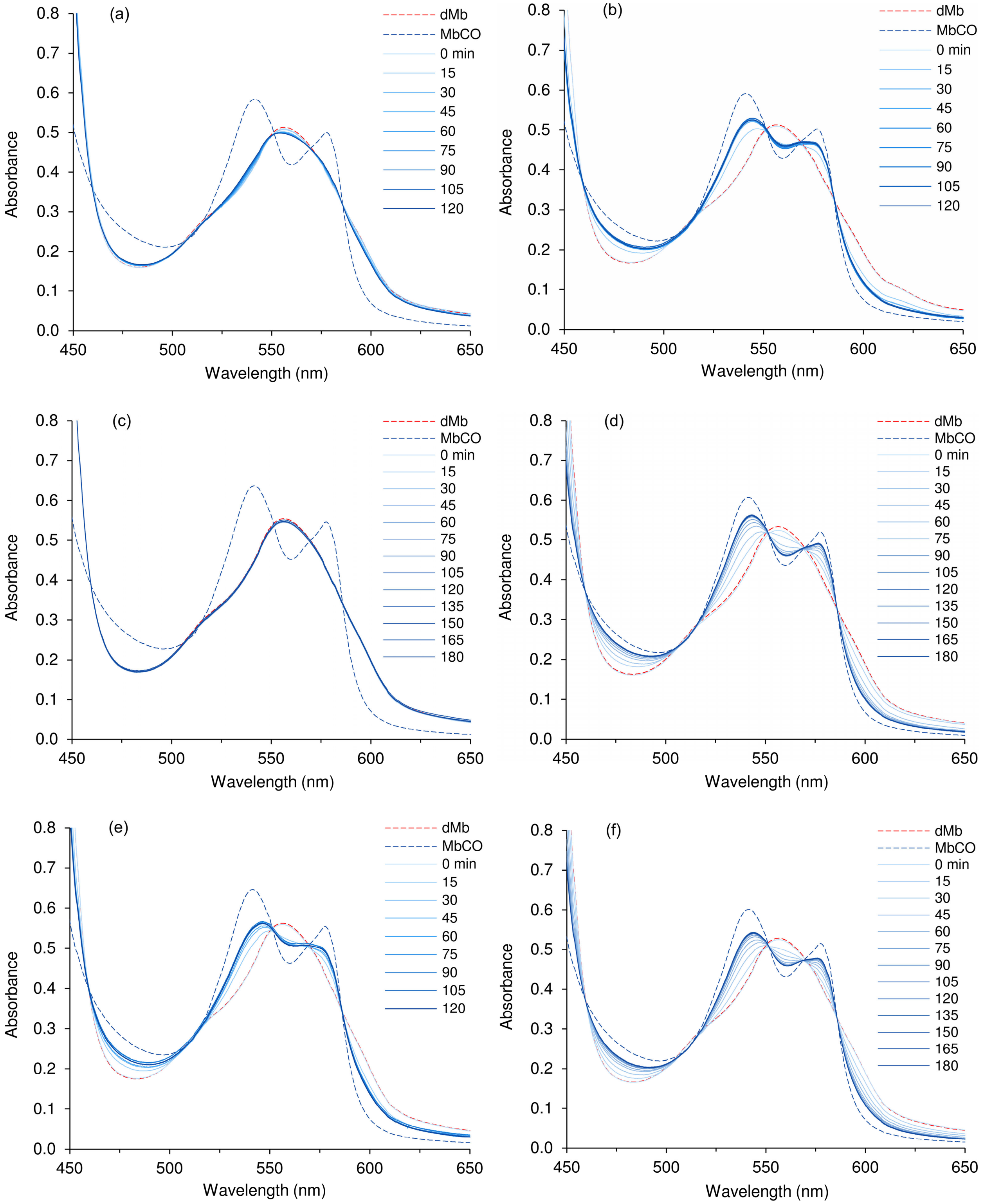

Fig. S20 UV-vis spectra for individual Mb assays performed with **1** (a,b), **2** (c,d), **1**@CB7 (e) and **2**@CB7 (f) either in the dark (a,c) or under visible light irradiation (b,e,f) (λ = 400-700 nm, E = 10 mW cm^{-2}, 37 °C, 0.01 M PBS, pH 7.4).

Table S1. Crystal data and structure refinement of 2@CB7

Formula	$C_{206.8}H_{209}Fe_4N_{116}O_{150.7}$
Formula weight	6954.09
Temperature / K	150(2)
Crystal system	Monoclinic
Space group	$P2_1/n$
a / Å	21.4035(6)
b / Å	20.5884(5)
c / Å	37.2781(8)
α / °	90.0
β / °	98.879(2)
γ / °	90.0
Volume / Å^3	16230.3(7)
Z	2
μ(Mo Kα) / mm^{-1}	0.291
Crystal type	White block
Crystal size / mm	0.23×0.18×0.13
θ range (°)	2.97 – 52.71
Index ranges	$-26 \leq h \leq 26$
	$-25 \leq k \leq 25$
	$-46 \leq l \leq 45$
Collected Reflections	196589
Independent Reflections	31237 (R_{int} = 0.1162)
Completeness to θ = 25.24	99.8%
Final R indices [$I > 2\sigma(I)$]	$R1$ = 0.1602
	$wR2$ = 0.4122
Final R indices (all data)	$R1$ = 0.2617
	$wR2$ = 0.4691
Largest diff. peak and hole /eÅ^{-3}	2.37 and −2.29
